# An artificial bee bare-bone hunger games search for global optimization and high-dimensional feature selection

**DOI:** 10.1016/j.isci.2023.106679

**Published:** 2023-04-21

**Authors:** Zhiqing Chen, Ping Xuan, Ali Asghar Heidari, Lei Liu, Chengwen Wu, Huiling Chen, José Escorcia-Gutierrez, Romany F. Mansour

**Affiliations:** 1 School of Intelligent Manufacturing, Wenzhou Polytechnic, Wenzhou 325035, China; 2 Department of Computer Science, School of Engineering, Shantou University, Shantou 515063, China; 3 Key Laboratory of Intelligent Informatics for Safety & Emergency of Zhejiang Province, Wenzhou University, Wenzhou 325035, China; 4 College of Computer Science, Sichuan University, Chengdu, Sichuan 610065, China; 5 Department of Computational Science and Electronics, Universidad de la Costa, CUC, Barranquilla 080002, Colombia; 6 Department of Mathematics, Faculty of Science, New Valley University, El-Kharga 72511, Egypt

**Keywords:** Genetics, Computational bioinformatics, Algorithms

## Abstract

The domains of contemporary medicine and biology have generated substantial high-dimensional genetic data. Identifying representative genes and decreasing the dimensionality of the data can be challenging. The goal of gene selection is to minimize computing costs and enhance classification precision. Therefore, this article designs a new wrapper gene selection algorithm named artificial bee bare-bone hunger games search (ABHGS), which is the hunger games search (HGS) integrated with an artificial bee strategy and a Gaussian bare-bone structure to address this issue. To evaluate and validate the performance of our proposed method, ABHGS is compared to HGS and a single strategy embedded in HGS, six classic algorithms, and ten advanced algorithms on the CEC 2017 functions. The experimental results demonstrate that the bABHGS outperforms the original HGS. Compared to peers, it increases classification accuracy and decreases the number of selected features, indicating its actual engineering utility in spatial search and feature selection.

## Highlights

• We propose a new method based on HGS with artificial bee and bare-bones strategies
• The balance and diversity analysis test validate the effect of two strategies on HGS
• The new method outperforms many state-of-the-art algorithms on CEC2017 functions
• This method performs well for feature selection on high-dimensional dataset

## Graphical abstract

**Figure:**
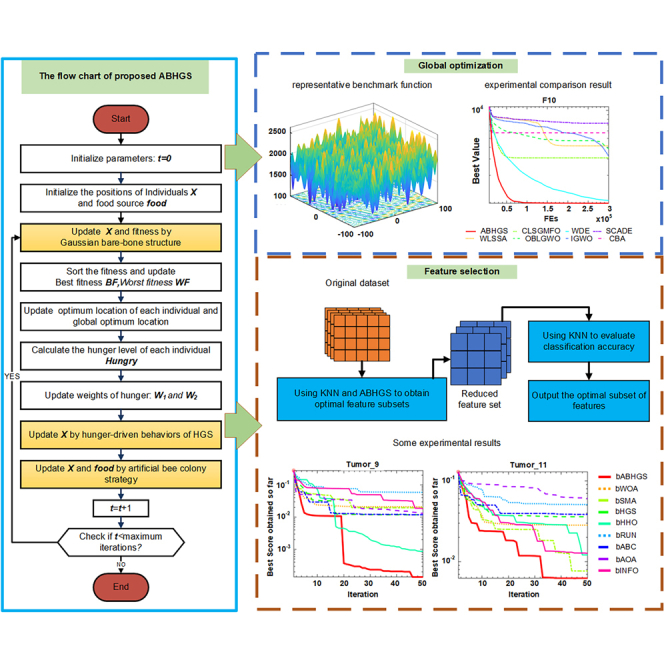

## Introduction

High-dimensional gene data classification analysis has received much attention in bioinformatics and computational biology, and it is possible to group genes according to specific structures, such as biological pathways. The expression of these genes in microarray technology^1^^,^^2^ is simultaneously analyzed and measured to help scholars understand disease at the genetic level. Selecting a set of related genes is preferable to choosing a single gene because selecting a gene ignores the information in the grouping structure is less efficient. Grouping effects indicate that strongly related genes tend to be chosen or not chosen together. For example, tens to hundreds of thousands of genes are measured from individuals in several experimental groups.^3^ Meanwhile, genetic expression data is highly dimensional and has extensive features.^4^^,^^5^ The unrelated and complex gene expression features reduce computational performance and waste resources.^6^^,^^7^^,^^8^ Gene selection is a feature selection (FS) in genes, which reduces unrelated genes and gene dimensions.^9^^,^^10^^,^^11^ Thus, the feature size of high-dimensional gene data becomes small, and classification performance can be improved effectively.^12^^,^^13^^,^^14^

Feature Selection (FS) is a process of determining the most relevant characteristics from a given dataset. By eliminating redundant and ineffective features, the number of features can be reduced to accelerate model training and enhance accuracy, especially in datasets with high dimensions.^15^^,^^16^ The model’s training grows increasingly challenging as the dataset’s features increase. The unnecessary features can increase the training time with worse performance in the model.^17^ Removing these superfluous features can add to the model’s success in feature selection before training. FS inevitably becomes a critical stage, especially in high-dimensional gene datasets, which is a helpful way to reduce redundant features.

Filter,^18^ wrapper,^19^ embedded,^20^ ensemble,^21^ and hybrid^22^^,^^23^ are the five main groups of feature selection approaches. Among them, wrapper methods rely on specific learning algorithms, such as classifiers, to explore a minimal number of feature subsets and can often achieve higher accuracy than filters.^24^^,^^25^ The search technique and the assessment criterion are the two main components of a wrapper design.^26^^,^^27^ In the former, a classifier, such as a support vector machine (SVM) or k-nearest neighbors (KNN),^28^ is used to evaluate the quality of the feature subset acquired during the search strategy module.^29^ In the latter, heuristic search is more efficient computationally than exhaustive and random search, and metaheuristic algorithms (MAs) can swiftly route to the ideal or nearly ideal solution.^30^

To find a high-quality solution in a limited time and build on it a mathematical model which maximizes or minimizes the objective function,^31^^,^^32^ in addition to traditional methods such as particle swarm optimization (PSO),^33^ some new algorithms have been proposed recently, including henry gas solubility optimization (HGSO),^34^ Archimedes optimization algorithm (AOA),^35^ honey badger algorithm (HBA),^36^ slime mold algorithm (SMA),^37^^,^^38^ Runge Kutta optimizer (RUN),^39^ colony predation algorithm (CPA),^40^ weighted mean of vectors (INFO),^41^ rime optimization algorithm (RIME),^42^ Harris hawks optimization (HHO)^43^ and so on. Recently, many hybrid algorithms have been devised and extensively implemented. For example, Celik^44^ proposed improved symbiotic organisms search (ISOS) algorithm for global optimization. Celik et al.^45^ propounded a modified salp swarm algorithm that outperformed the original (SSA) algorithm and many recent algorithms. Houssein et al.^46^ advocated an improved sooty tern optimization algorithm to solve the feature selection problem. Celik^47^ proposed an information-exchanged Gaussian arithmetic optimization algorithm with quasi-opposition learning to solve optimization problems. These algorithms have shown some superiority in different fields such as bankruptcy prediction,^48^ scheduling optimization,^49^^,^^50^ economic emission dispatch,^51^ multi-objective optimization,^52^ feedforward neural networks,^53^ dynamic multi-objective optimization,^54^ large-scale complex optimization,^55^ constrained multi-objective optimization,^56^ global optimization,^57^ and feature selection.^58^^,^^59^^,^^60^

In addition, several new hybrid metaheuristic algorithms (MAs) have been developed to address the feature selection issue. Hammouri et al.^61^ devised a binary dragonfly algorithm (BDA) by utilizing several ways to update the values of five essential coefficients for feature selection. In summary, the proposed updating technique significantly impacts the algorithm’s ability to solve FS problems. Adding chaotic maps to the original population led Tahir et al.^62^ to develop a binary chaotic genetic algorithm (BCGA). Affective database AMIGOS and two healthcare datasets with huge feature spaces evaluate the novel BCGA with standard GA and two other state-of-the-art approaches. A new method named OBSSO for social spider optimization (SSO) using an OBL (opposition-based learning) technique was suggested by Ibrahim et al.^63^ The accuracy of OBSSO was then compared to that of the standard SSO, the artificial bee colony (ABC), the firefly algorithm (FA), and the sine cosine algorithm (SCA) across ten datasets using a combination of KNN and RF classifiers. An improved salp swarm algorithm (ISSA) was suggested by Tubishat et al.^64^ to locate the ideal feature subset using the opposition-based learning approach (OBL)and a new local search algorithm (LSA). On 18 datasets, ISSA’s performance was examined and contrasted with four traditional methods. Too et al. proposed a robust hyper-learning binary dragonfly algorithm (HLBDA).^27^ The hyper-learning technique was devised to assist DA in breaking out of the local optimum and enhancing the search behavior. To assess the success of their alterations, twenty-one datasets were employed, one of which relates to the coronavirus disease (COVID-19).

In the research above, metaheuristic strategies have outperformed feature selection approaches.^65^ Despite its advantages, MAs have certain drawbacks in practice, such as the risk of getting stuck in local optimum, sub-optimal solutions, and slow convergence rate.^43^^,^^66^^,^^67^ From this point of view, a more efficient optimizer is needed to identify the ideal set of dataset features. To reach this goal, a high-performance metaheuristic called Hunger Games Search (HGS) is chosen and used for feature selection in this research for at least the following reasons. Firstly, compared to other optimizers, especially those based on animal behavior,^68^ HGS not only takes inspiration from an animal with a particular behavior but also creates a universal metaphor that implies the survival rules of nature. Yang et al.,^69^ conducted simulations which showed that HGS outperformed six established and nine modern MAs on 23 benchmark functions. Furthermore, HGS was also more successful than nine enhanced algorithms and seven DE variants algorithms on the IEEE CEC 2014 test suite. HGS has been employed to adjust the parameters of a hybrid microgrid system^70^ as well as to create a new soft computing model for predicting the intensity of ground vibrations caused by mine blasting.^71^ HGS has proven its excellence in AI through its impressive performance in terms of solution quality and computing cost, thereby highlighting its superiority.

Thus, HGS has been adopted by researchers to solve optimization issues. For example, an advanced orthogonal learning and Gaussian barebone hunger games search method were proposed by Zhou et al. for engineering optimization problems.^72^ A hunger search-based whale optimization algorithm (HSWOA), as an ensemble of HGS and WOA,^73^ was presented by Chakraborty et al. for global optimization.^74^ Li et al. proposed a novel hybrid HGS with DE, chaotic local search, and evolutionary population dynamics technique, denoted as DECEHGS, for engineering designs and global optimization.^75^ A new HGS-based algorithm optimized the multiple layers perceptron neural network to decrease the error.^76^ A Laplacian Nelder-Mead HGS, named LNMHGS, was proposed by Yu et al. to optimize the parameter of photovoltaic technology.^77^ Devi et al. presented two binary versions of HGS according to the V transfer function and S transfer function within a wrapper feature selection method for selecting features from low and medium datasets.^78^ Houssein et al. presented a modified HGS to improve the support vector machine (SVM) classification performance for feature selection using chemical and medical datasets.^79^ Ma et al. introduced a multi-strategy to HGS, and the binary version was applied to reduce data dimensionality, which can be a valuable wrapper feature selection method.^80^ In addition, Devi et al.^78^ used HGS in the process of feature selection. During this process, the researchers investigated both V-shaped and S-shaped transfer functions. However, they only compared HGS against five different classical algorithms and one more advanced algorithm.

The above studies show that although HGS exhibits superior characteristics, it still faces the probability of local stagnation due to its strong development ability. Moreover, the hybrid algorithm performs better in solving problems such as feature selection tasks. Thus, to improve the global exploration and local exploitation search, this article proposes a modified HGS with an artificial bee strategy and a Gaussian bare-bone, namely ABHGS. To validate the superiority of ABHGS, we designed a set of experiments. The HGS with the operator of ABC is named AHGS. The Gaussian bare-bone modified HGS, abbreviated as BHGS. After adding the mechanism, the balance analysis of ABHGS, AHGS, BHGS, and HGS shows the different changes in global exploration and local exploitation. Besides, ABHGS is also performed on CEC2017 test functions to compare with AHGS, BHGS, and original HGS. The results prove that ABHGS outperforms the others significantly in this article. To evaluate the effectiveness of ABHGS, we apply 14 public UCI high-dimensional gene classification datasets and compare them with other competitors. Consequently, ABHGS gains a new balance of global explorative and local exploitative strategies to achieve satisfactory performance. Experimental results and statistical tests show the excellent performance of ABHGS.

In a word, the main contribution of this article is listed as follows.

• An artificial bee strategy and a Gaussian bare-bone structure are introduced to HGS, and an enhanced algorithm ABHGS is developed to promote the tradeoff between exploration and exploitation.
• The history trajectory and balance analysis show the excellent performance of the ABHGS.
• The ABHGS compares against many conventional and advanced algorithms on the IEEE CEC2017 test function.
• The binary ABHGS-KNN model ranks first in high-dimensional gene selection problems compared with other state-of-the-art methods.

## Results and discussion

To validate the effectiveness of ABHGS, sets of experiments are designed. To prove the cooperation of artificial bee colony strategy and Gaussian bare-bone structure, we created HGS with artificial bee colony strategy, which is named AHGS, and HGS with Gaussian bare-bone structure BHGS. The overall experiments are conducted in the same hardware and MATLAB R2018b software environment. The hardware is a computer with the CPU of 12th Gen Intel (R) Core (TM) I7-12700H (2.30 GHz) Windows 11 data edge of 16.0 GB RAM.

A historical search trajectory test of ABHGS is conducted, as described in the history trajectory section. Balance analysis and diversity analysis section shows the balance analysis and diversity analysis among ABHGS, AHGS, BHGS, and HGS, showing the new balance between global exploration and local exploitation. Then ABHGS compares with AHGS, BHGS, and HGS on CEC2017 test functions to prove its global optimization capacity in the verification of the mechanisms section. ABHGS outperforms the other six conventional algorithms on the CEC2017 functions in the comparative test with conventional metaheuristic algorithms section. Furthermore, the enhanced algorithms are compared with ABHGS on thirty CEC2017 test functions to illustrate its excellent performance in comparative test with several modified algorithms section. The feature selection section shows that ABHGS conducts 14 University of California Irvine (UCI) machine learning repositories for high-dimensional gene selection.

Thirty CEC2017 benchmark functions are chosen to validate the effectiveness of ABHGS. Four types of functions exist in the thirty IEEE CEC2017 test functions set, including unimodal functions (F1-F3), basic multimodal functions (F4-F10), hybrid functions (F11-F20), and composite functions (F21-F30). *F(min)* is the only global optimal solution with a boundary range. The single-peak function evaluates the local exploitation capacity. By contrast, multimodal functions are suitable for benchmarking detection abilities for global exploration capacity. Besides, composite functions take into account the balance of local exploitation and global exploration at the same time.

### History trajectory

**Figure 1:**
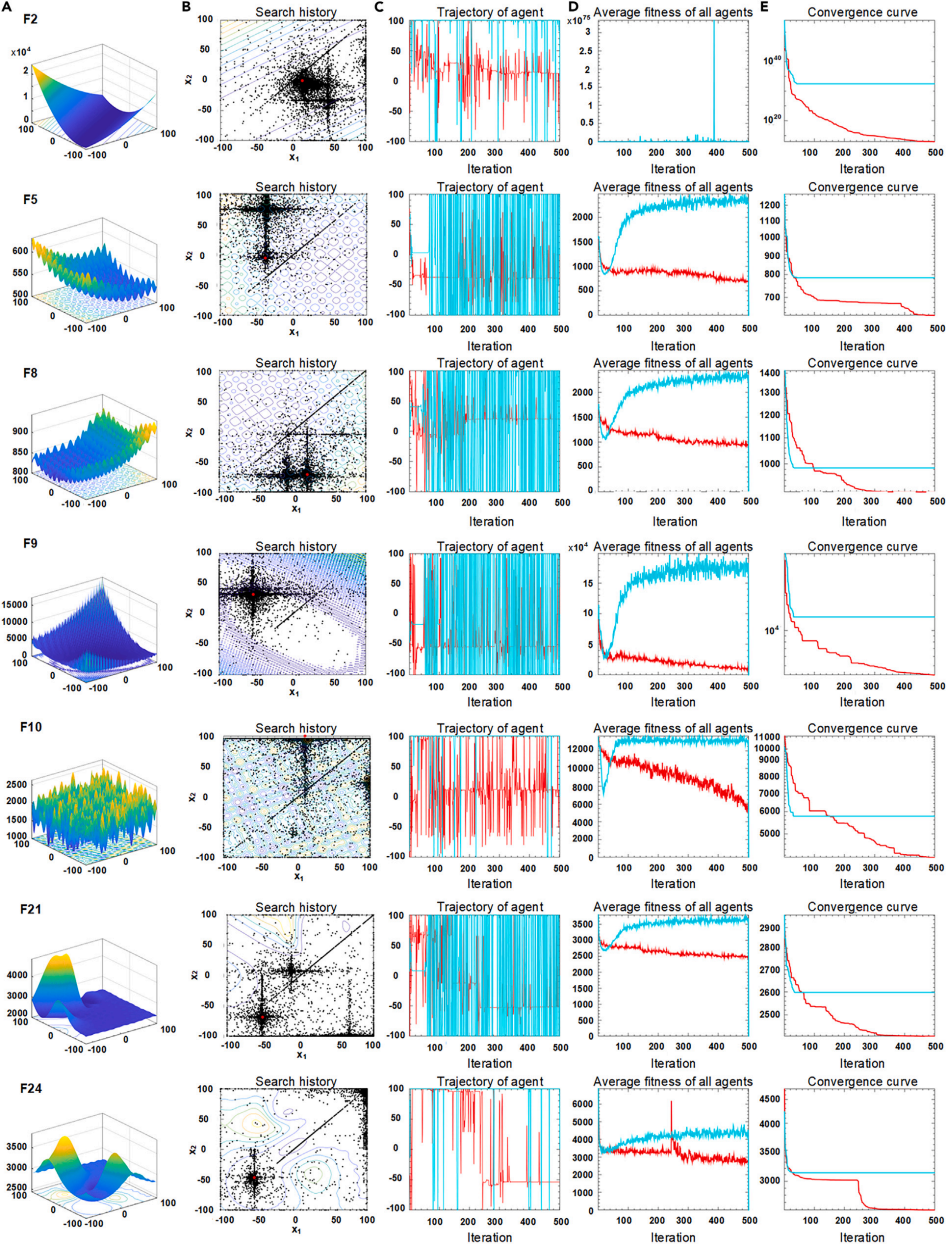
The history trajectory analysis for ABHGS and HGS (A) Graphical plots of functions. (B) Search history of ABHGS. (C) Trajectory of ABHGS and HGS in the first dimension. (D) The average fitness of ABHGS and HGS. (E) The convergence curves of ABHGS and HGS.

This section contains several historical trajectories of functions for ABHGS to assess the impact of local exploitation and global exploration on optimized performance.^92^^,^^93^ Figure 1 shows the historical trajectory of four types of CEC2017 of test functions in 500 iterations, including a unimodal function F2, four basic multimodal functions F5, F8, F9, and F10, and two composite functions, F21 and F24. Figure 1A shows the three-dimensional location distribution. In Figure 1B, the red dots represent the global optimum solution, and the black dots represent positions in every iteration. In Figures 1C, 1D, and 1(c), the red line is ABHGS, and the other line represents HGS. Figure 1A describes the graphical plots of the selected mathematical functions based on ABHGS. Figure 1B shows the individual’s historical search of the ABHGS method on the mentioned functions in 500 iterations, distributing around the best solution in the search space. Figure 1C presents the trajectory obtained by ABHGS with iterations in the first dimension. It shows the fluctuating state of the individual to gain the best value. Besides, ABHGS is volatile in the early period but becomes stable later. This phenomenon indicates a high probability of the population spreading around the optimal point. However, for HGS, individuals whose value fluctuates up and down are likelier to stay in the global explorative search stage than in the local exploitative search stage. Figure 1D illustrates the average fitness of agents with iterations. As can be seen from the curve, the mean fitness of ABHGS with fast convergence obtains the minimal value of final convergence. Figure 1E is the curve of functional fitness calculated by ABHGS and HGS. Obviously, ABHGS gains the final optimized minimal value with fast convergence.

In brief, there are the history trajectory graphs of seven representative functions: F2, F5, F8, F9, F10, F21, and F24. It shows the graphical plot and analyzes the search history of ABHGS on the functions. Besides, the comparisons about the trajectories of agents, the average fitness of all agents, and the convergence curves in ABHGS and HGS are shown in Figure 1. In conclusion, ABHGS is superior to HGS in unimodal, multimodal, and composition functions. ABHGS, with a relatively large proportion of global exploration searches in the early stage, always seeks the optimal solution. The capacity of ABHGS to find the best solution is stronger than that of HGS. Therefore, ABHGS gets a smaller convergence value and faster convergence speed than HGS.

### Balance analysis and diversity analysis

In this section, further balance analysis of the exploration and exploitation of ABHGS, AHGS, BHGS, and HGS can help us better understand the reason for excellent performance in global optimization cases. The parameter setting in the main function is the same. For example, the maximal evaluation number is set to 300,000. The population size is 30, and each experiment runs 30 times independently. To calculate the increase and decrease in the distance among search agents, a diversity measurement known as the dimension-wise diversity measurement is calculated by Equations 1 and 2 in each iteration.

(Equation 1) $${\mathrm{DIV}}_{j}=\frac{1}{N}\sum_{i=1}^{N}\left|median\left(X^{j}\right)-X_{i}^{j}\right|$$

(Equation 2) $$\mathrm{DIV}=\frac{1}{D}\sum_{j=1}^{D}{\mathrm{DIV}}_{j}$$

(Equation 3) $${\mathrm{DIV}}_{\max}=\max\left\{{\mathrm{DIV}}^{1},{\mathrm{DIV}}^{2},\ldots ,{\mathrm{DIV}}^{t},\ldots ,{\mathrm{DIV}}^{Maxiter}\right\}$$

where $median\left(X^{j}\right)$ represents the median of dimension j in the whole population. $X_{i}^{j}$ is the dimension j of search agent i. N corresponds to the number of search agents in the population while D symbolizes the dimension of the search agents. t represents the current iteration. The highest diversity value identified during the entire optimization process is ${\mathrm{DIV}}_{\max}$. The detailed pseudo-code of diversity calculation is presented in Algorithm 1.

Algorithm 1 **Pseudo-code of diversity calculation**

**INPUT：** The hungry search agents X,N,D(dimension);

 **While** (t ≤Maxiter)

 For i=1:N

 For j=1:D

 Calculate the diversity in each dimension ${\mathrm{DIV}}_{j}$ by Equation (1);

 End

 Calculate the diversity of the entire population DIV by averaging every ${\mathrm{DIV}}_{j}$ in each dimension using Equation (2);

 End

 
t=t+1;

 
**End While**

 Calculate the highest diversity value ${\mathrm{DIV}}_{\max}$ using Equation (3);

**OUTPUT**$\mathrm{DIV},{\mathrm{DIV}}_{\max}$;

The percentage of exploration and exploitation is used to describe the total balance response. Equation (4) and Equation (5) are used to calculate these values in each iteration.

(Equation 4) $$Exploration\%=\left(\frac{\mathrm{DIV}}{DIV_{\max}}\right)*100$$

(Equation 5) $$Exploitation\%=\left(\frac{\left|DIV_{\max}-\mathrm{DIV}\right|}{DIV_{\max}}\right)*100$$

In this model, The Exploration% denotes the degree of exploration, which is the ratio between the diversity in each iteration and the maximum attainable diversity. On the other hand, Exploitation% is a complementary percentage to Exploration%, as it reflects the difference between the maximum diversity and the current diversity of an iteration, which clustering search agents cause.

**Figure 2:**
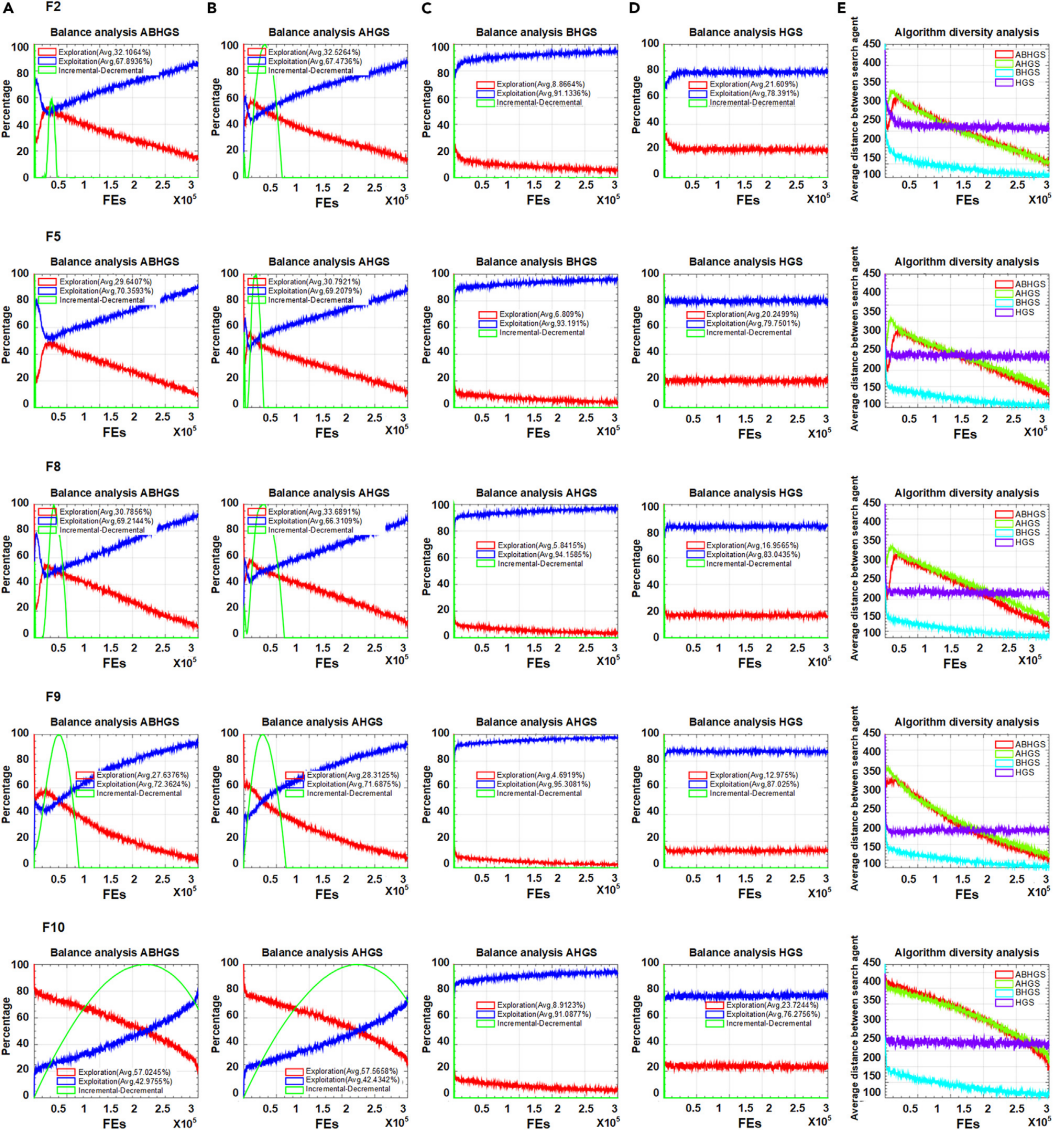
The balance and diversity analysis on F2,F5,F8,F9,F10 (A) Balance analysis of ABHGS. (B) Balance analysis of AHGS. (C) Balance analysis of BHGS. (D) Balance analysis of HGS. (E) Diversity analysis of algorithms (F2, F5, F8, F9, F10).

**Figure 3:**
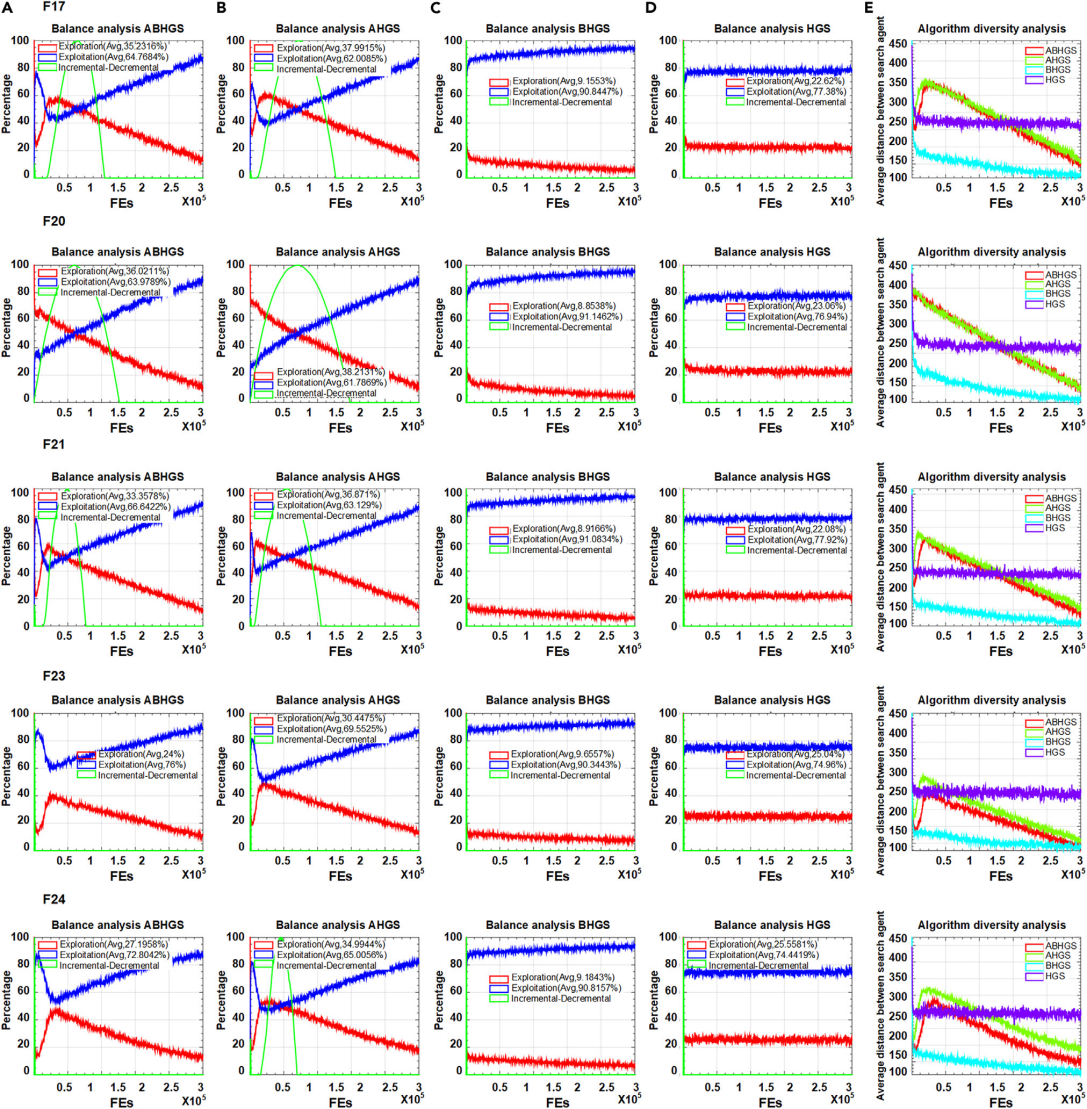
The balance and diversity analysis on F17,F20,F21,F23,F24 (A) Balance analysis of ABHGS. (B) Balance analysis of AHGS. (C) Balance analysis of BHGS. (D) Balance analysis of HGS. (E) Diversity analysis of algorithms (F17, F20, F21, F23, F24).

To assess the artificial bee colony strategy and Gaussian bare-bone structure independently, we design AHGS and BHGS. Through adding the mechanism, the explore and exploit capacities are affected. There is synergy between the different changes. Theoretical and experimental justifications about the effect of modifications on the proposed algorithm can be illustrated and discussed. Figures 2 and 3 exhibits the balance analyses of ABHGS, AHGS, BHGS, and HGS and their diversity analyses on 10 functions selected from the CEC2017 test functions, including F2, F5, F8, F9, F10, F17, F20, F21, F23, and F24. As can be seen from Figures 2 and 3, the balance of ABHGS, AHGS, BHGS, and HGS and their diversity are analyzed. There are exploration, exploitation, and incremental-decremental curves in the balance analysis of ABHGS, AHGS, BHGS, and HGS. Red lines indicate global exploration search, and blue curves show local exploitation search. Green lines represent the incremental-decremental curve. AHGS has a larger proportion of global exploration search and a smaller proportion of local exploitation search than HGS.

However, BHGS has a more significant percentage of local exploitation search and a lower rate of global exploration search than HGS. Such experimental results are because of the addition of different mechanisms. One mechanism can expand global search capability, and the other can strengthen local search capability. Inspired by this idea, we combine these two mechanisms to HGS and develop ABHGS, which may lead to a new equilibrium. The experimental results confirm that the concept and the proportion of exploration and exploitation of ABHGS fall between AHGS and BHGS, which is different from HGS. Then, if the exploration search is larger than the exploitation search, the green curve shows an upward trend. A downward trend is presented if the exploration search is less than or equal to the exploitation search. The duration with low or high values with iteration in the figure reflects persistent outcomes of local exploitation or global exploration capabilities in the search strategy. The ten functions evaluated by ABHGS have a higher exploration proportion than HGS, exhibiting its extensive global exploration effects. HGS has a relatively low exploration percentage and strong local exploitation search ability. The decline curve is the largest with the iterations when diversity and intensification are at the same level. To avoid HGS falling into local optima with the stagnation of convergence, ABHGS continues the global exploration phase.

The diversity analysis of mentioned F2, F5, F8, F9, F10, F17, F20, F21, F23, and F24 functions are shown in the last line of Figures 2 and 3. The x axis is the iteration number, while the y axis specifies the diversity measure. Algorithms always start with great diversity because the initialization is random. As the number of iterations increases, the population diversity gradually decreases. The results show that the variety of HGS does not reduce after reaching a value. The diversity values of ABHGS decline faster and are smaller than HGS. The diversity curve indicates that ABHGS converges faster and is earlier in global exploration than HGS.

### Verification of the mechanisms

After the above analysis and validation, ABHGS was compared to AHGS, BHGS, and HGS on the IEEE CEC2017 test functions. Besides, the influence of artificial bee colony strategy and Gaussian bare-bone structure are investigated on the optimization issues. Analyzing the mathematical model’s elements and their verification and numerical values is crucial to showing its working logic. The entire method was tested in the same software and hardware environment for a fair comparison. The parameter setting in the primary function is the same; for example, the maximal evaluation number is set to 300,000. The population size is 30, and each experiment is run 30 times independently. These methods evaluated their performance using the statistical average value of the optimal function (Avg) and SD(Std). The best result obtained by algorithms for each function in the table is highlighted in bold. Of course, the smaller the value, the better the performance. If the modification is considered significant statistically, the Wilcoxon signed-rank test was less than 0.05; that is, the p value is less than 0.05. The Wilcoxon signed-rank test is a non-parametric statistical test at a significance level of 0.05. The Friedman test is a statistical conformance test, too. The symbols “+/ = /-” illustrate that the proposed algorithm performs better, equal, or worse than the other comparative method.

**Figure 4:**
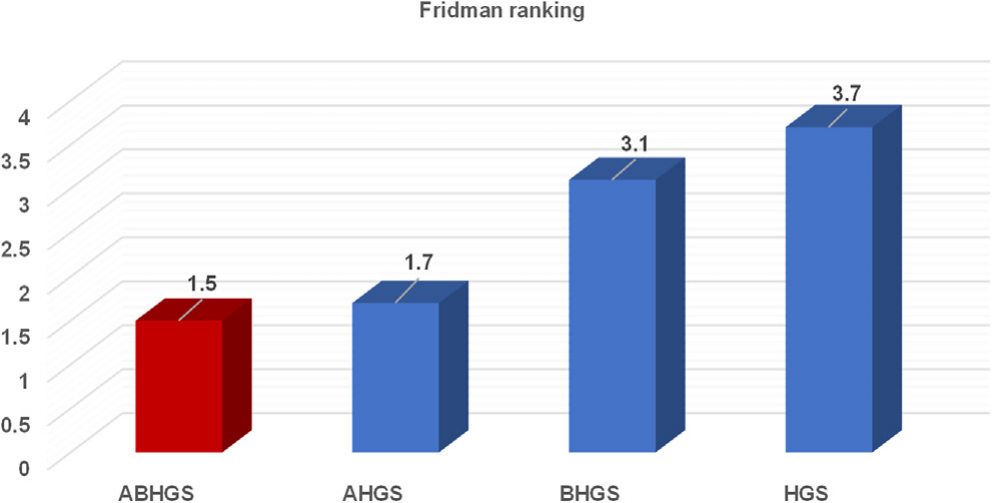
Friedman ranking of ABHGS, AHGS, BHGS, and HGS

Comparative tests of ABHGS, AHGS, BHGS, and HGS on IEEE CEC2017 are executed to validate the excellent performance of ABHGS. Table 1 displays the statistical average (Avg) and SD(Std) of the involved methods for each function independently. The minimal values in every row of Table 1 are marked in bold. Table 2 shows that the p value computed by the Wilcoxon signed-rank test and the values less than 0.05 are observed in bold. It also displays the average ranking result (AVR) value and rank results by the Wilcoxon signed-rank test. Figure 4 shows the Friedman ranking test of ABHGS, AHGS, BHGS, and HGS. From Tables 2 and 3 and Figure 4, the experimental results indicate that ABHGS performs better than AHGS, BHGS, and HGS because an artificial bee colony strategy enhances the global exploration of HGS significantly, and a Gaussian bare-bone improves the local exploitation of HGS in arriving at the optimal solution. As shown in Figure 4 ABHGS has an average 1.5 ranking value by the Friedman test, which is superior to AHGS, BHGS, and HGS. The results show that ABHGS with an artificial bee colony strategy and a Gaussian bare-bone structure can perform well. Therefore, the artificial bee colony strategy and Gaussian bare-bone design significantly affect the performance of HGS.

**Table 1:** Comparative results for ABHGS, AHGS, BHGS, and HGS

Function	Metric	ABHGS	AHGS	BHGS	HGS
F1	Avg	**3.9851E+02**	9.4864E+02	4.0937E+05	1.1934E+08
	Std	**4.9152E+02**	1.3686E+03	8.2990E+05	1.3258E+08
F2	Avg	4.2759E+07	**1.0961E+05**	4.7675E+17	7.0308E+20
	Std	2.1061E+08	**5.7809E+05**	2.4522E+18	1.5424E+21
F3	Avg	1.8414E+03	2.1330E+03	**6.8258E+02**	4.4479E+03
	Std	7.5834E+02	9.8006E+02	**2.0039E+03**	8.5838E+03
F4	Avg	**4.4044E+02**	4.3679E+02	4.9434E+02	5.0161E+02
	Std	3.2287E+01	**3.1837E+01**	9.0558E+01	3.6395E+01
F5	Avg	**5.9575E+02**	5.9779E+02	6.2612E+02	6.1996E+02
	Std	**1.6841E+01**	1.9995E+01	3.6306E+01	2.8975E+01
F6	Avg	**6.0000E+02**	**6.0000E+02**	6.0010E+02	6.0250E+02
	Std	3.4366E-13	**4.2222E-14**	2.2717E-01	1.1781E+00
F7	Avg	**8.1000E+02**	8.1140E+02	8.7002E+02	8.7174E+02
	Std	1.4469E+01	**1.2667E+01**	4.8175 E+01	4.1653E+01
F8	Avg	**8.9834E+02**	9.0167E+02	9.0650E+02	9.1336E+02
	Std	**1.5621E+01**	1.7392E+01	2.3113E+01	2.8034E+01
F9	Avg	**1.8125E+03**	2.0016E+03	3.1910E+03	3.4541E+03
	Std	5.3907E+02	**4.5149E+02**	9.0498E+02	1.0983E+03
F10	Avg	**3.4555E+03**	3.4663E+03	3.9522E+03	4.0464E+03
	Std	**3.2742E+02**	3.4910E+02	5.9120E+02	4.4779E+02
F11	Avg	**1.1796E+03**	1.2023E+03	1.1996E+03	1.2523E+03
	Std	**3.5661E+01**	3.4969E+01	3.9451E+01	1.2034E+02
F12	Avg	3.7280E+05	**2.5625E+05**	3.0900E+06	2.5444E+06
	Std	3.9298E+05	**1.8183E+05**	5.2022E+06	1.4777E+06
F13	Avg	**6.9360E+03**	7.2927E+03	2.1946E+04	3.7803E+04
	Std	7.3013E+03	**6.0836E+03**	3.0149E+04	2.6133E+04
F14	Avg	4.4452E+04	**4.4080E+04**	5.3562E+04	4.6259E+04
	Std	4.7968E+04	**4.3400E+04**	5.7094E+04	4.1769E+04
F15	Avg	**2.5519E+03**	2.9832E+03	1.0018E+04	2.2203E+04
	Std	**1.1986E+03**	2.5396E+03	9.9406E+03	1.6839E+04
F16	Avg	**2.2993E+03**	2.3596E+03	2.6769E+03	2.6884E+03
	Std	**2.3555E+02**	1.6448E+02	2.1137E+02	1.8446E+02
F17	Avg	**1.9620E+03**	2.0159E+03	2.2441E+03	2.2801E+03
	Std	**1.3316E+02**	1.3172E+02	1.6009E+02	2.1172E+02
F18	Avg	**1.5275E+05**	1.3455E+05	1.5590E+05	2.6344E+05
	Std	**1.1166E+05**	6.1719E+04	1.2716E+05	2.1918E+05
F19	Avg	**3.3478E+03**	2.8856E+03	1.1737E+04	2.0015E+04
	Std	**1.7242E+03**	1.3343E+03	1.4393E+04	2.1235E+04
F20	Avg	**2.3244E+03**	2.3348E+03	2.5203E+03	2.5177E+03
	Std	1.4420E+02	**1.1215E+02**	1.9014E+02	1.8566E+02
F21	Avg	2.3717E+03	**2.3716E+03**	2.4227E+03	2.4294E+03
	Std	7.7357E+01	7.7164E+01	**2.5876E+01**	3.3655E+01
F22	Avg	**3.5319E+03**	4.1399E+03	5.3152E+03	4.7555E+03
	Std	**1.3663E+03**	1.4666E+03	1.3516E+03	1.4489E+03
F23	Avg	2.7399E+03	**2.7318E+03**	2.7740E+03	2.7684E+03
	Std	2.5042E+01	3.4687E+01	2.8941E+01	**2.4664E+01**
F24	Avg	2.9394E+03	**2.9292E+03**	3.0012E+03	3.0335E+03
	Std	1.4014E+02	1.7960E+02	6.0931E+01	**5.1080E+01**
F25	Avg	**2.8792E+03**	2.8843E+03	2.8911E+03	2.8894E+03
	Std	1.5035E+00	**1.3953E+00**	1.8409E+01	9.7243E+00
F26	Avg	3.6804E+03	**3.5923E+03**	4.6771E+03	4.9350E+03
	Std	9.6375E+02	1.0296E+03	**4.2263E+02**	6.3717E+02
F27	Avg	**3.2000E+03**	3.2129E+03	**3.2000E+03**	3.2252E+03
	Std	3.5950E-04	7.5570E+00	**3.1248E-04**	1.6108E+01
F28	Avg	3.1865E+03	**3.1787E+03**	3.2872E+03	3.2631E+03
	Std	**2.7111E+01**	3.2269E+01	2.8365E+01	5.1986E+01
F29	Avg	**3.5195E+03**	3.6404E+03	3.7275E+03	3.7841E+03
	Std	1.3856E+02	**1.3755E+02**	2.0186E+02	1.7472E+02
F30	Avg	**6.2031E+03**	8.6824E+03	7.5464E+03	7.6487E+04
	Std	3.3437E+03	**2.2712E+03**	8.0427E+03	1.0098E+05

**Table 2:** p value results of Wilcoxon test

Function	ABHGS	AHGS	BHGS	HGS
F1	∼	1.414E-01	**1.734E-06**	**1.734E-06**
F2	∼	**1.957E-02**	**1.238E-05**	**1.734E-06**
F3	∼	3.086E-01	**3.112E-05**	3.933E-01
F4	∼	5.304E-01	**5.706E-04**	**1.921E-06**
F5	∼	7.189E-01	**3.589E-04**	**3.162E-03**
F6	∼	**7.098E-06**	**1.734E-06**	**1.734E-06**
F7	∼	4.779E-01	**2.127E-06**	**2.127E-06**
F8	∼	5.038E-01	1.414E-01	**4.070E-02**
F9	∼	1.915E-01	**9.316E-06**	**1.238E-05**
F10	∼	5.170E-01	**9.711E-05**	**1.973E-05**
F11	∼	**2.304E-02**	8.972E-02	**7.514E-05**
F12	∼	2.369E-01	**1.149E-04**	**1.734E-06**
F13	∼	6.288E-01	**9.842E-03**	**2.370E-05**
F14	∼	9.590E-01	5.857E-01	8.612E-01
F15	∼	4.653E-01	**2.643E-04**	**4.286E-06**
F16	∼	3.086E-01	**1.025E-05**	**9.316E-06**
F17	∼	1.254E-01	**1.025E-05**	**1.238E-05**
F18	∼	6.733E-01	8.130E-01	**8.307E-04**
F19	∼	1.589E-01	**4.114E-03**	**1.150E-04**
F20	∼	8.130E-01	**2.052E-04**	**1.114E-03**
F21	∼	9.918E-01	**2.255E-03**	**4.897E-04**
F22	∼	5.984E-02	**3.881E-04**	**1.593E-03**
F23	∼	3.389E-01	**8.919E-05**	**1.287E-03**
F24	∼	2.712E-01	1.846E-01	**8.944E-04**
F25	∼	**1.734E-06**	**8.307E-04**	**1.734E-06**
F26	∼	8.290E-01	**1.742E-04**	**3.724E-05**
F27	∼	**2.353E-06**	4.653E-01	**1.734E-06**
F28	∼	4.528E-01	**1.734E-06**	**1.734E-06**
F29	∼	**4.390E-03**	**2.225E-04**	**7.691E-06**
F30	∼	**3.379E-03**	8.451E-01	**2.353E-06**
+/−/ =	∼	5/2/23	21/1/7	28/0/2
ARV	**1.5**	1.7	3.1	3.7
Rank	**1**	2	3	4

**Table 3:** Specified parameters of involved MAs

Method	Reference	Common parameters	Parameters
RSA	Abualigah et al.^129^	Dimension = dim; population size = 30	a=0.1;β=0.1
INFO	Ahmadianfar et al.^41^	Dimension = dim; population size = 30	c = 2; d = 4
CPA	Tu et al.^40^	Dimension = dim; population size = 30	$a=\exp\left(9-18*\frac{fes}{Max_{iter}}\right);S0=a*\left(1-\frac{fes}{Max_{iter}}\right)$
SMA	Kumar et al.^130^	Dimension = dim; population size = 30	z=0.03;
AOA	Hashim et al.^35^	Dimension = dim; population size = 30	a=5
HGS	Yang et al.^69^	Dimension = dim; population size = 30.	l=0.08; LH=100;
RUN	Ahmadianfar et al.^39^	Dimension = dim; population size = 30.	ɡ=[02];
HHO	Heidari et al.^43^	Dimension = dim; population size = 30.	∼

**Figure 5:**
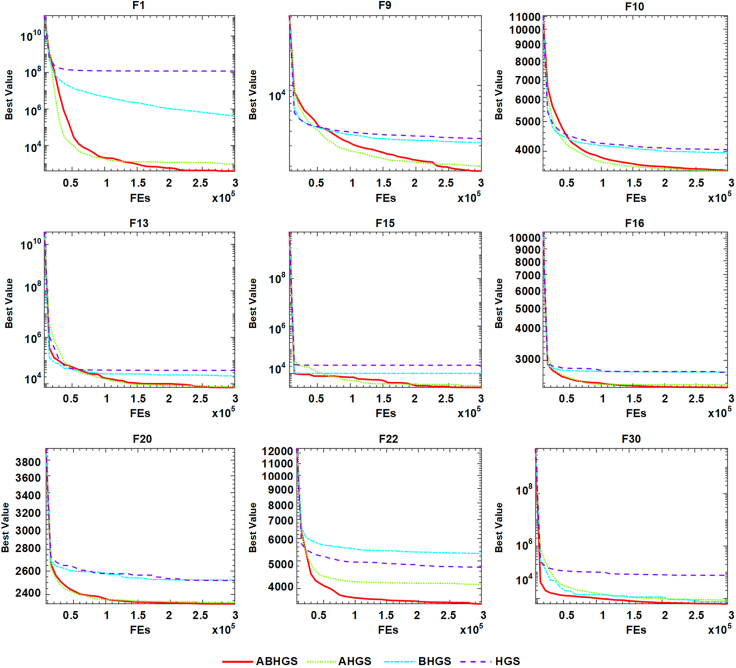
The fitness convergence curve of ABHGS, AHGS, BHGS, and HGS on CEC2017 functions

Convergence curves of the comparison of ABHGS, AHGS, BHGS, and HGS on the CEC2017 test function are presented in Figure 5. There are F1, F9, F10, F13, F15, F16, F20, F22 and F30. As is shown in Figure 5, ABHGS has a satisfactory effect after using two strategies compared with the basic HGS. Combining artificial bee colony strategy and Gaussian bare-bone structure assists HGS in arriving at global optima and avoiding the optimal local solution. The advantage of ABHGS is significant, and the effect of the two mechanisms on HGS is positive. Therefore, ABHGS has a better-optimized ability than AHGS, BHGS, and HGS.

### Comparative test with conventional metaheuristic algorithms

**Figure 6:**
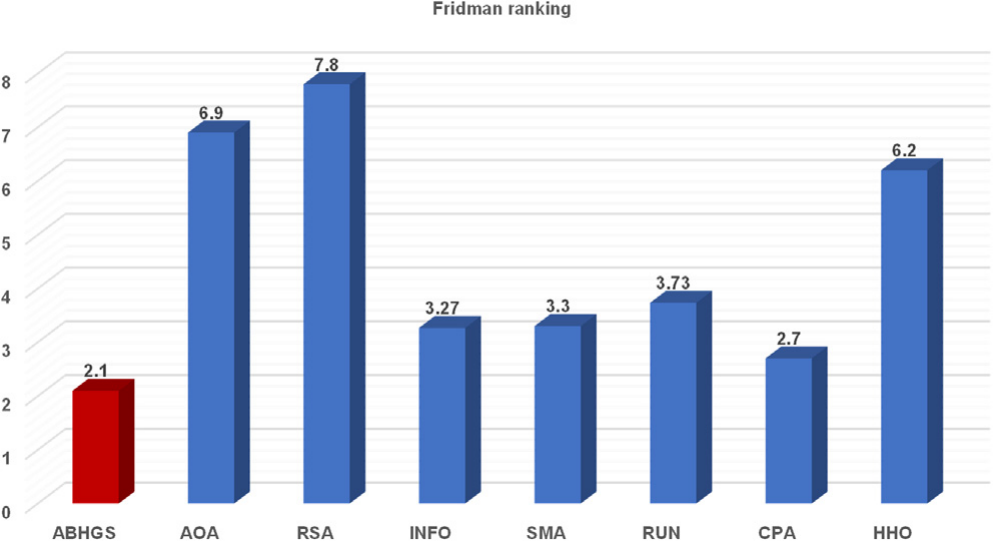
Friedman ranking of ABHGS and other conventional algorithms

**Figure 7:**
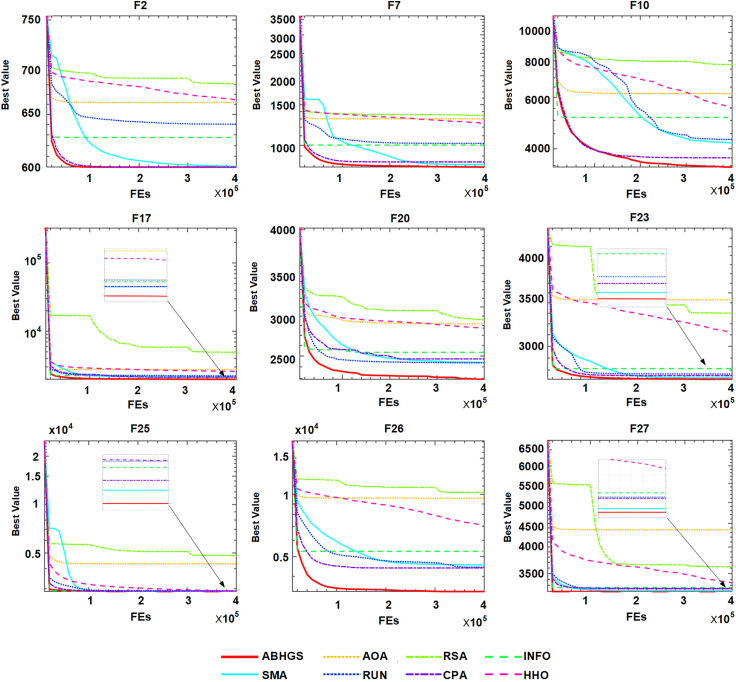
Convergence curves of ABHGS and other conventional algorithms on CEC2017

To validate the global exploration search and local exploitation search abilities of ABHGS, it is compared with some typical conventional algorithms, such as AOA, RSA, NIFO, SMA, CPA, HHO, and RUN. Table 3 lists the specified parameters of the involved MAs. In this section, we conduct IEEE CEC2017 benchmark functions to show ABHGS’s global exploration search ability. The simulation results of the involved methods show that ABHGS converges quickly and reaches the minimum, which demonstrates the excellent global exploration and local exploitation performance of ABHGS. Tables 4 and 5 record the mean value, std value, p value, and rank test ARV of the simulation experiment. Figure 6 describes the rank test ARV of the Friedman test. Figure 7 presents convergence curves of the mentioned algorithms on the CEC2017 function test set.

**Table 4:** Comparative results for ABHGS and conventional methods

Function	Metric	ABHGS	AOA	RSA	INFO	SMA	RUN	CPA	HHO
F1	Avg	3.9810E+02	4.0775E+10	5.0719E+10	**1.0000E+02**	8.0012E+03	5.5017E+03	3.0553E+03	1.0923E+07
	Std	4.3564E+02	6.3059E+09	9.1416E+09	**6.2328E-07**	7.2639E+03	5.1138E+03	3.5534E+03	2.0634E+06
F2	Avg	1.0323E+10	7.6289E+39	1.0074E+49	**2.0000E+02**	2.0000E+02	2.0000E+02	2.0000E+02	3.4432E+12
	Std	4.9265E+10	2.5637E+40	5.5177E+49	1.6278E-04	8.3860E-04	1.8751E-03	**1.2298E-04**	6.0400E+12
F3	Avg	1.2314E+03	7.2936E+04	7.3465E+04	3.0000E+02	3.0001E+02	3.0016E+02	**3.0000E+02**	6.1088E+03
	Std	7.5985E+02	6.4483E+03	7.0930E+03	2.1260E-06	3.2557E-03	1.0993E-01	**6.2703E-08**	2.5752E+03
F4	Avg	4.3697E+02	9.3010E+03	9.1447E+03	**4.1901E+02**	4.8916E+02	4.9572E+02	4.7739E+02	5.1699E+02
	Std	3.0668E+01	2.3311E+03	2.8591E+03	2.6981E+01	**4.5538E+00**	1.9813E+01	3.6006E+01	2.9020E+01
F5	Avg	**5.9492E+02**	7.8462E+02	8.8625E+02	6.6263E+02	5.8556E+02	6.9296E+02	6.2663E+02	7.3507E+02
	Std	1.3332E+01	3.9574E+01	3.2394E+01	3.8323E+01	**2.2880E+01**	4.2970E+01	2.3186E+01	2.1184E+01
F6	Avg	**6.0000E+02**	6.6189E+02	6.8063E+02	6.2758E+02	6.0066E+02	6.4038E+02	6.0000E+02	6.6411E+02
	Std	**3.6566E-14**	6.0042E+00	4.8908E+00	1.2328E+01	3.5771E-01	7.9130E+00	3.1313E-13	4.7206E+00
F7	Avg	**8.0656E+02**	1.3035E+03	1.3466E+03	1.0040E+03	8.2637E+02	1.0211E+03	8.4887E+02	1.2371E+03
	Std	**1.2867E+01**	4.9641E+01	3.5585E+01	6.8557E+01	2.7793E+01	6.4883E+01	2.9508E+01	9.2178E+01
F8	Avg	8.9446E+02	1.0329E+03	1.1165E+03	9.2553E+02	**8.9377E+02**	9.4086E+02	9.1014E+02	9.5815E+02
	Std	1.9604E+01	3.4495E+01	1.8293E+01	2.9843E+01	2.0117E+01	1.9559E+01	**1.8156E+01**	1.9789E+01
F9	Avg	**1.6379E+03**	5.7891E+03	9.0245E+03	3.0541E+03	1.7918E+03	3.4946E+03	2.0427E+03	6.7180E+03
	Std	**5.2200E+02**	8.0971E+02	6.7816E+02	8.4061E+02	1.1899E+03	7.0198E+02	6.0542E+02	6.5717E+02
F10	Avg	**3.4596E+03**	6.1803E+03	7.7658E+03	5.1203E+03	4.1865E+03	4.3027E+03	3.7221E+03	5.5101E+03
	Std	3.5398E+02	5.3622E+02	**3.5369E+02**	7.2765E+02	4.9660E+02	7.0596E+02	5.6339E+02	6.7284E+02
F11	Avg	1.1978E+03	2.7152E+03	9.8559E+03	1.2647E+03	1.2419E+03	**1.2105E+03**	1.1656E+03	1.2547E+03
	Std	2.9354E+01	7.4958E+02	4.0998E+03	6.0835E+01	5.1157E+01	2.7940E+01	**3.4083E+01**	5.2505E+01
F12	Avg	3.8659E+05	6.9606E+09	1.3969E+10	**1.5376E+04**	6.7588E+05	1.6359E+06	1.4477E+06	1.2243E+07
	Std	3.3795E+05	2.6751E+09	2.9376E+09	**1.2040E+04**	7.2484E+05	6.5162E+05	8.6320E+05	7.5027E+06
F13	Avg	4.6563E+03	3.6616E+04	8.4995E+09	1.5437E+04	3.7989E+04	2.8218E+04	**3.2846E+03**	3.5472E+05
	Std	3.4895E+03	1.9703E+04	4.8482E+09	1.5355E+04	2.6187E+04	1.4765E+04	**2.6374E+03**	1.4670E+05
F14	Avg	4.8554E+04	5.0016E+04	3.4564E+06	**1.6821E+03**	2.7695E+04	2.0966E+03	5.7639E+03	6.4944E+04
	Std	4.9526E+04	4.5845E+04	3.2693E+06	**1.4735E+02**	1.1824E+04	5.3348E+02	4.2297E+03	6.1755E+04
F15	Avg	2.8218E+03	2.2830E+04	6.5334E+08	**2.1482E+03**	2.8274E+04	1.5884E+04	2.3808E+03	6.5841E+04
	Std	2.3401E+03	1.0216E+04	5.2347E+08	**1.4069E+03**	1.4877E+04	1.8926E+03	1.6554E+03	6.4084E+04
F16	Avg	**2.3452E+03**	4.2016E+03	5.2867E+03	2.6272E+03	2.4462E+03	2.5882E+03	2.7776E+03	3.2956E+03
	Std	**1.7993E+02**	8.8824E+02	6.6832E+02	2.3534E+02	3.3221E+02	2.5141E+02	2.7537E+02	3.6363E+02
F17	Avg	**1.9816E+03**	2.7492E+03	4.9393E+03	2.1988E+03	2.1188E+03	2.2266E+03	2.1268E+03	2.5728E+03
	Std	**1.1873E+02**	3.0402E+02	2.4649E+03	2.3043E+02	1.5910E+02	2.2719E+02	1.7981E+02	2.5994E+02
F18	Avg	1.7712E+05	9.8687E+05	1.9148E+07	**6.1098E+03**	2.4021E+05	3.6518E+04	9.0185E+04	1.0194E+06
	Std	1.1573E+05	1.9282E+06	1.5557E+07	**4.3290E+03**	1.4897E+05	1.0199E+04	4.3198E+04	1.0218E+06
F19	Avg	2.9533E+03	9.8164E+05	6.6452E+08	**2.0511E+03**	3.8106E+04	7.5945E+03	4.3938E+03	3.5783E+05
	Std	1.1329E+03	1.4090E+05	4.8184E+08	**8.7783E+01**	2.1936E+04	2.6057E+03	1.8904E+03	2.4947E+05
F20	Avg	**2.2916E+03**	2.8179E+03	2.8618E+03	2.5341E+03	2.4409E+03	2.4366E+03	2.4727E+03	2.7720E+03
	Std	1.3861E+02	1.6203E+02	**1.2859E+02**	2.2575E+02	1.6255E+02	1.3137E+02	1.4711E+02	2.1913E+02
F21	Avg	2.3818E+03	2.5950E+03	2.6926E+03	2.4419E+03	**2.3980E+03**	2.4176E+03	2.3666E+03	2.5596E+03
	Std	6.0983E+01	5.7675E+01	4.4609E+01	4.3404E+01	2.7878E+01	5.4545E+01	**9.0888E+01**	4.6583E+01
F22	Avg	3.5868E+03	**8.0737E+03**	8.1615E+03	4.0381E+03	5.5729E+03	3.1879E+03	3.2942E+03	6.8623E+03
	Std	1.5164E+03	8.0465E+02	8.7267E+02	2.2150E+03	8.8766E+02	**1.6852E+03**	1.5686E+03	1.3533E+03
F23	Avg	**2.7258E+03**	3.4305E+03	3.3016E+03	2.8116E+03	2.7378E+03	2.7676E+03	2.7549E+03	3.1232E+03
	Std	2.5012E+01	1.3251E+02	9.2572E+01	5.9055E+01	**2.2718E+01**	3.2125E+01	2.7235E+01	1.3364E+02
F24	Avg	2.9417E+03	3.8115E+03	3.4221E+03	2.9937E+03	2.9120E+03	**2.9086E+03**	3.0984E+03	3.4363E+03
	Std	1.3294E+02	1.8587E+02	2.0178E+02	4.0493E+01	2.7644E+01	**2.3036E+01**	8.0959E+01	1.2075E+02
F25	Avg	**2.8787E+03**	4.2801E+03	4.8210E+03	2.9043E+03	2.8881E+03	2.9087E+03	2.8951E+03	2.9091E+03
	Std	**1.3603E+00**	5.0677E+02	6.6594E+02	2.1498E+01	8.8167E+00	1.8575E+01	1.2490E+01	2.0857E+01
F26	Avg	**3.3790E+03**	9.5881E+03	1.0232E+04	5.2966E+03	4.5516E+03	4.4346E+03	4.3972E+03	7.0246E+03
	Std	8.6770E+02	6.8052E+02	6.4722E+02	1.2782E+03	**1.7247E+02**	1.5512E+03	1.2701E+03	1.2954E+03
F27	Avg	**3.2000E+03**	4.3561E+03	3.6226E+03	3.2626E+03	3.2118E+03	3.2487E+03	3.2449E+03	3.3418E+03
	Std	**3.1067E-04**	4.3049E+02	1.2942E+02	3.3201E+01	1.0166E+01	2.1455E+01	1.8700E+01	8.1051E+01
F28	Avg	3.1640E+03	5.9060E+03	6.2529E+03	3.1320E+03	**3.2232E+03**	3.1061E+03	3.1436E+03	3.2527E+03
	Std	4.1254E+01	6.7342E+02	6.1454E+02	5.5435E+01	**6.0976E+01**	2.3241E+01	5.4180E+01	2.6936E+01
F29	Avg	3.4734E+03	**6.2768E+03**	5.8812E+03	4.1145E+03	3.7902E+03	4.0827E+03	3.6642E+03	4.3937E+03
	Std	1.1740E+02	**8.9327E+02**	6.0943E+02	2.8256E+02	1.8040E+02	1.8068E+02	1.9629E+02	2.7337E+02
F30	Avg	**5.6379E+03**	7.3597E+07	2.5042E+09	6.0925E+03	1.7034E+04	6.9570E+04	1.2663E+04	1.5206E+06
	Std	2.5485E+03	1.9628E+08	1.2780E+09	**8.2618E+02**	4.3799E+03	4.6411E+04	4.7869E+03	7.9473E+05

**Table 5:** p value of Wilcoxon test of involved conventional algorithms

Function	ABHGS	AOA	RSA	INFO	SMA	RUN	CPA	HHO
F1	∼	**1.7344E-06**	**1.7344E-06**	**1.7344E-06**	**2.6033E-06**	**1.9209E-06**	**4.2857E-06**	**1.7344E-06**
F2	∼	**1.7344E-06**	**1.7344E-06**	**1.7344E-06**	**1.7344E-06**	**1.7344E-06**	**1.7344E-06**	**1.7344E-06**
F3	∼	**1.7344E-06**	**1.7344E-06**	**1.7344E-06**	**1.7344E-06**	**1.7344E-06**	**1.7344E-06**	**1.9209E-06**
F4	∼	**1.7344E-06**	**1.7344E-06**	**5.3197E-03**	**1.7344E-06**	**5.2165E-06**	**2.2248E-04**	**1.7344E-06**
F5	∼	**1.7344E-06**	**1.7344E-06**	**1.7344E-06**	7.1903E-02	**2.1266E-06**	**6.9838E-06**	**1.7344E-06**
F6	∼	**1.7344E-06**	**1.7344E-06**	**1.7344E-06**	**1.7344E-06**	**1.7344E-06**	**7.2378E-08**	**1.7344E-06**
F7	∼	**1.7344E-06**	**1.7344E-06**	**1.7344E-06**	**4.6818E-03**	**1.7344E-06**	**8.4661E-06**	**1.7344E-06**
F8	∼	**1.7344E-06**	**1.7344E-06**	**2.2248E-04**	8.1302E-01	**1.9209E-06**	**5.3197E-03**	**1.7344E-06**
F9	∼	**1.7344E-06**	**1.7344E-06**	**2.6033E-06**	8.2901E-01	**1.9209E-06**	**2.4308E-02**	**1.7344E-06**
F10	∼	**1.7344E-06**	**1.7344E-06**	**1.7344E-06**	**2.3704E-05**	**3.4053E-05**	**3.0010E-02**	**1.7344E-06**
F11	∼	**1.7344E-06**	**1.7344E-06**	**5.3070E-05**	**1.0357E-03**	1.5886E-01	**7.1570E-04**	**4.4493E-05**
F12	∼	**1.7344E-06**	**1.7344E-06**	**1.7344E-06**	6.5641E-02	**4.2857E-06**	**2.1630E-05**	**1.7344E-06**
F13	∼	**1.7344E-06**	**1.7344E-06**	**7.5137E-05**	**6.9838E-06**	**1.7344E-06**	**4.7162E-02**	**1.7344E-06**
F14	∼	7.9710E-01	**1.7344E-06**	**1.7344E-06**	2.2888E-01	**1.7344E-06**	**3.1817E-06**	1.1093E-01
F15	∼	**1.7344E-06**	**1.7344E-06**	5.4463E-02	**3.1817E-06**	**1.7344E-06**	5.9994E-01	**1.7344E-06**
F16	∼	**1.7344E-06**	**1.7344E-06**	**3.1123E-05**	2.0589E-01	**8.3071E-04**	**1.7988E-05**	**1.7344E-06**
F17	∼	**1.7344E-06**	**1.7344E-06**	**9.7110E-05**	**2.0515E-04**	**1.3601E-05**	**3.3173E-04**	**1.7344E-06**
F18	∼	**6.8923E-05**	**1.7344E-06**	**1.7344E-06**	7.5213E-02	**2.1266E-06**	**1.0357E-03**	**6.3198E-05**
F19	∼	**1.7344E-06**	**1.7344E-06**	**4.0715E-05**	**2.3534E-06**	**1.7344E-06**	**4.5336E-04**	**1.7344E-06**
F20	∼	**1.7344E-06**	**1.7344E-06**	**4.4493E-05**	**1.1138E-03**	**3.6094E-03**	**2.2248E-04**	**1.7344E-06**
F21	∼	**1.7344E-06**	**1.7344E-06**	**3.1123E-05**	6.7328E-01	**8.7297E-03**	4.1653E-01	**1.7344E-06**
F22	∼	**1.9209E-06**	**1.7344E-06**	2.4519E-01	**3.8822E-06**	7.0356E-01	6.1431E-01	**2.1630E-05**
F23	∼	**1.7344E-06**	**1.7344E-06**	**3.8822E-06**	**3.1603E-02**	**5.3070E-05**	**3.5888E-04**	**1.7344E-06**
F24	∼	**1.7344E-06**	**1.7344E-06**	8.5896E-02	**4.1140E-03**	**3.6094E-03**	**6.9838E-06**	**1.7344E-06**
F25	∼	**1.7344E-06**	**1.7344E-06**	**1.7344E-06**	**1.7344E-06**	**1.7344E-06**	**1.7344E-06**	**1.7344E-06**
F26	∼	**1.7344E-06**	**1.7344E-06**	**8.4661E-06**	**5.3070E-05**	**5.3070E-05**	**3.3789E-03**	**2.3534E-06**
F27	∼	**1.7344E-06**	**1.7344E-06**	**1.7344E-06**	**3.8822E-06**	**1.7344E-06**	**1.7344E-06**	**1.7344E-06**
F28	∼	**1.7344E-06**	**1.7344E-06**	**2.1827E-02**	**3.3173E-04**	**1.3601E-05**	1.9152E-01	**1.7344E-06**
F29	∼	**1.7344E-06**	**1.7344E-06**	**1.7344E-06**	**6.3391E-06**	**1.7344E-06**	**1.4773E-04**	**1.7344E-06**
F30	∼	**1.7344E-06**	**1.7344E-06**	1.2044E-01	**2.3534E-06**	**1.7344E-06**	**7.6909E-06**	**1.7344E-06**
+/−/ =	∼	29/0/1	30/0/0	17/9/4	19/3/8	22/6/2	20/6/4	29/0/1
ARV	**2.1**	6.9	7.8	3.266667	3.3	3.733333	2.7	6.2
Rank	**1**	7	8	3	4	5	2	6

As can be seen from Table 4, the minimum value in each function is marked in bold. Though ABHGS has not gained the smallest mean value on F1, F2, F3, F4, F8, F11, F12, F13, F14, F15, F18, F19, F21, F22, F24, F28, and F29, it outperforms the other swarm intelligent algorithms in handling the rest functions. Nevertheless, ABHGS ranks first in the Wilcoxon test in the whole functions in Table 5. The most p value of the Wilcoxon test is less than 0.05, which shows statistical significance. Figure 6 shows the results of the Friedman ranking test and ABHGS with the value of 2.1, ranking first. The results validate the excellent performance of ABHGS when compared with the conventional algorithms involved. ABHGS shows improved global exploration ability compared to the conventional algorithms involved.

Figure 7 presents the convergence curve of the above algorithms on the CEC2017 test functions. There are F6, F7, F10, F17, F20, F23, F25, F26, and F27. We can intuitively discover that ABHGS converges fast and finds the optima accurately. The combination of artificial bee colony strategy and Gaussian bare-bone structure enables HGS to arrive at a higher quality solution in the optimization process, achieving a better equilibrium between global exploration and local exploitation.

### Comparative test with several modified algorithms

**Figure 8:**
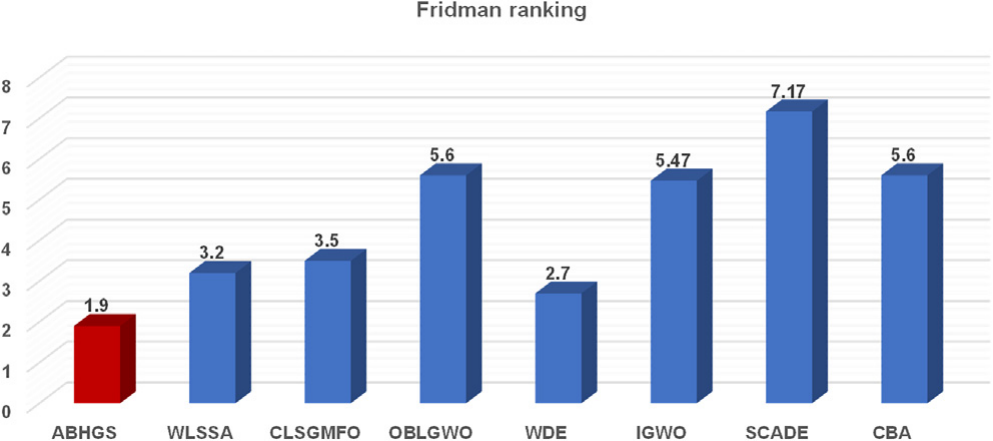
Friedman ranking of advanced algorithms

From the previous analysis, the advantages of the ABHGS algorithm should be further verified on CEC 2017 benchmark functions, compared with several state-of-the-art advanced algorithms. These modified algorithms are adaptive weights levy-assisted SSA (WLSSA),^94^ chaotic mutative MFO (CLSGMFO),^95^ efficient boosted GWO (OBLGWO),^96^ weighted DE (WDE),^97^ improved GWO (IGWO),^98^ the hybrid algorithm of SCA and DE (SCADE),^99^ cloud bat algorithm (CBA).^100^ Tables 6 and 7 show the experimental results between ABHGS and eight advanced algorithms on CEC 2017 functions. The minimum of each test function was bold. Table 6 illustrates the comparison’s mean value and SD value for each algorithm. As can be seen from Table 7, the statistical outcomes show the p value of the Wilcoxon signed-rank test result among advanced swarm intelligent algorithms. Most p values are less than 0.05. Figure 8 describes the Friedman ranking test of advanced algorithms and shows the superiority of ABHGS. The Freidman test value gained by ABHGS is 1.9, also displayed in Table 7, ranking it first. WDE also performs well, ranking second. ABHGS outperforms involved advanced algorithms on CEC 2017 functions. Therefore, ABHGS can be superior to some advanced competitors.

**Table 6:** Comparative results for ABHGS and involved improved methods

Function	Metric	ABHGS	WLSSA	CLSGMFO	OBLGWO	WDE	IGWO	SCADE	CBA
F1	Avg	4.5371E+06	4.1599E+06	1.0441E+06	1.4490E+07	**6.0611E+05**	1.5317E+07	4.6623E+08	3.9466E+06
	Std	4.0070E+06	2.9102E+06	4.4952E+05	5.4860E+06	**2.0366E+05**	5.5935E+06	1.1130E+08	1.5811E+06
F2	Avg	**2.0220E+02**	1.2926E+04	1.8299E+04	1.5997E+07	1.1056E+04	2.3930E+06	2.9369E+10	5.4052E+03
	Std	**5.7492E+00**	9.2775E+03	1.5166E+04	7.9415E+06	3.4292E+03	9.2201E+05	2.1750E+09	7.5436E+03
F3	Avg	3.4819E+02	1.3935E+04	3.3211E+03	8.5586E+03	**3.0062E+02**	6.5980E+03	5.7256E+04	3.4943E+03
	Std	5.6135E+01	3.5461E+03	2.1718E+03	4.2945E+03	**1.2730E-01**	2.6626E+03	6.2827E+03	3.5776E+03
F4	Avg	**4.5851E+02**	5.0411E+02	4.8820E+02	5.3829E+02	4.6619E+02	5.2209E+02	2.3130E+03	5.1176E+02
	Std	2.7744E+01	2.7053E+01	4.2206E+01	3.5881E+01	**2.0217E+01**	2.6494E+01	3.4449E+02	3.1698E+01
F5	Avg	**5.2001E+02**	5.2006E+02	5.2013E+02	5.2093E+02	5.2007E+02	5.2056E+02	5.2095E+02	5.2019E+02
	Std	**1.0619E-02**	1.1654E-01	2.2841E-01	4.4394E-02	1.8664E-02	1.5418E-01	8.2171E-02	2.1994E-01
F6	Avg	6.1512E+02	**6.1211E+02**	6.1835E+02	6.1992E+02	6.1608E+02	6.1873E+02	6.3396E+02	6.3958E+02
	Std	**1.9686E+00**	4.8382E+00	3.1784E+00	4.5945E+00	2.0843E+00	4.6154E+00	3.2930E+00	3.3318E+00
F7	Avg	**7.0001E+02**	7.0001E+02	7.0001E+02	7.0117E+02	7.0003E+02	7.0099E+02	9.0732E+02	7.0001E+02
	Std	1.2272E-02	1.3624E-02	1.5276E-02	8.4477E-02	7.3337E-03	3.3212E-02	2.7376E+01	**7.0421E-03**
F8	Avg	**8.0000E+02**	9.2019E+02	9.0626E+02	9.3667E+02	8.1444E+02	8.9604E+02	1.0791E+03	9.9760E+02
	Std	**0.0000E+00**	1.8351E+01	1.9631E+01	3.4934E+01	2.0663E+00	2.6998E+01	1.6404E+01	3.8737E+01
F9	Avg	**9.9156E+02**	1.0523E+03	1.0644E+03	1.0753E+03	1.0016E+03	1.0221E+03	1.2033E+03	1.1409E+03
	Std	2.2061E+01	1.8499E+01	2.3469E+01	3.7097E+01	**1.0560E+01**	2.1359E+01	1.6667E+01	3.9741E+01
F10	Avg	**1.0001E+03**	4.1492E+03	3.0774E+03	3.8483E+03	1.0730E+03	3.2145E+03	7.2658E+03	5.7336E+03
	Std	**4.3374E-02**	5.8246E+02	7.9686E+02	1.1296E+03	2.8717E+01	2.7433E+02	4.7398E+02	5.7496E+02
F11	Avg	**3.2137E+03**	4.7824E+03	4.9573E+03	5.1664E+03	3.3326E+03	4.3699E+03	8.1299E+03	5.9026E+03
	Std	2.5590E+02	7.2628E+02	4.5888E+02	8.7875E+02	2.0409E+02	5.3983E+02	**1.8732E+02**	5.9126E+02
F12	Avg	**1.2001E+03**	1.2003E+03	1.2006E+03	1.2023E+03	1.2002E+03	1.2008E+03	1.2024E+03	1.2008E+03
	Std	4.2672E-02	2.0172E-01	2.3823E-01	6.4085E-01	**2.5443E-02**	2.8272E-01	3.4312E-01	2.4416E-01
F13	Avg	**1.3002E+03**	1.3006E+03	1.3005E+03	1.3005E+03	1.3003E+03	1.3006E+03	1.3038E+03	1.3006E+03
	Std	4.5625E-02	7.2601E-02	1.1273E-01	1.4051E-01	**2.8036E-02**	1.2265E-01	3.0739E-01	1.6596E-01
F14	Avg	1.4002E+03	1.4003E+03	1.4004E+03	1.4004E+03	**1.4002E+03**	1.4004E+03	1.4821E+03	1.4004E+03
	Std	3.0530E-02	2.7203E-01	1.4541E-01	9.5443E-02	**2.2072E-02**	2.8687E-01	1.5760E+01	5.4197E-02
F15	Avg	1.5078E+03	**1.5056E+03**	1.5123E+03	1.5163E+03	1.5124E+03	1.5160E+03	2.0767E+04	1.5667E+03
	Std	1.7413E+00	1.8582E+00	4.7768E+00	5.4633E+00	**1.3463E+00**	3.8937E+00	8.5647E+03	2.0207E+01
F16	Avg	**1.6100E+03**	1.6117E+03	1.6111E+03	1.6121E+03	1.6108E+03	1.6115E+03	1.6127E+03	1.6133E+03
	Std	5.0282E-01	5.6421E-01	5.5223E-01	5.2887E-01	3.9169E-01	6.6316E-01	**2.6806E-01**	3.6133E-01
F17	Avg	6.8385E+05	2.4492E+05	3.2083E+05	1.7436E+06	**4.5846E+03**	8.0971E+05	1.5416E+07	1.8500E+05
	Std	4.3135E+05	1.6450E+05	2.6786E+05	1.5380E+06	**4.2882E+02**	6.6332E+05	6.2715E+06	7.3326E+04
F18	Avg	2.4035E+03	3.3824E+03	5.4231E+03	6.1144E+04	**1.8471E+03**	1.5551E+04	1.7760E+08	9.7128E+03
	Std	6.6959E+02	1.9249E+03	4.5291E+03	4.7716E+04	**9.6772E+00**	1.6103E+04	9.1320E+07	9.0338E+03
F19	Avg	**1.9078E+03**	1.9117E+03	1.9118E+03	1.9115E+03	1.9081E+03	1.9165E+03	2.0197E+03	1.9362E+03
	Std	**5.4663E-01**	2.3024E+00	1.9971E+00	2.0430E+00	6.9854E-01	3.1085E+00	1.0499E+01	2.9118E+01
F20	Avg	2.8807E+03	4.1461E+03	3.7781E+03	6.0731E+03	**2.0574E+03**	3.2119E+03	2.2230E+04	2.9323E+03
	Std	6.2073E+02	2.6925E+03	6.5517E+02	3.9530E+03	**1.0849E+01**	7.8804E+02	8.1134E+03	7.9257E+02
F21	Avg	1.6855E+05	1.2042E+05	1.5366E+05	5.3825E+05	**3.0415E+03**	3.4780E+05	2.1425E+06	1.0649E+05
	Std	8.1186E+04	1.2348E+05	7.4708E+04	3.8815E+05	**1.7157E+02**	3.3520E+05	7.5918E+05	7.5248E+04
F22	Avg	2.5229E+03	2.5952E+03	2.8174E+03	2.6971E+03	**2.3704E+03**	2.5468E+03	3.1103E+03	3.6172E+03
	Std	1.5768E+02	1.1518E+02	2.6841E+02	2.2998E+02	**7.4130E+01**	1.5820E+02	1.4602E+02	4.7847E+02
F23	Avg	**2.5000E+03**	**2.5000E+03**	**2.5000E+03**	2.6180E+03	2.6152E+03	2.6232E+03	2.5000E+03	2.6158E+03
	Std	**0.0000E+00**	**0.0000E+00**	**0.0000E+00**	1.6424E+00	1.0448E-03	5.0280E+00	0.0000E+00	2.0545E-01
F24	Avg	2.6000E+03	**2.6000E+03**	**2.6000E+03**	**2.6000E+03**	2.6304E+03	2.6000E+03	2.6000E+03	2.6732E+03
	Std	1.3559E-04	**0.0000E+00**	**0.0000E+00**	**0.0000E+00**	7.9858E-01	3.6014E-03	8.6774E-12	3.0940E+01
F25	Avg	**2.7000E+03**	**2.7000E+03**	**2.7000E+03**	**2.7000E+03**	2.7066E+03	2.7114E+03	**2.7000E+03**	2.7336E+03
	Std	**0.0000E+00**	**0.0000E+00**	**0.0000E+00**	**0.0000E+00**	8.9818E-01	1.7135E+00	**0.0000E+00**	1.7794E+01
F26	Avg	2.7004E+03	2.7005E+03	2.7204E+03	2.7006E+03	**2.7003E+03**	2.7007E+03	2.7040E+03	2.7006E+03
	Std	1.0423E-01	1.3440E-01	4.1978E+01	1.2776E-01	**3.0708E-02**	1.2392E-01	4.3507E-01	2.6175E-01
F27	Avg	**2.9000E+03**	**2.9000E+03**	**2.9000E+03**	3.0308E+03	3.1145E+03	3.1098E+03	3.2403E+03	3.9659E+03
	Std	**0.0000E+00**	**0.0000E+00**	**0.0000E+00**	2.7646E+02	5.0675E+00	3.8850E+00	2.4778E+02	4.6142E+02
F28	Avg	**3.0000E+03**	**3.0000E+03**	**3.0000E+03**	3.5172E+03	3.7751E+03	3.8123E+03	4.9111E+03	4.9477E+03
	Std	**0.0000E+00**	**0.0000E+00**	**0.0000E+00**	5.5931E+02	3.1176E+01	1.9283E+02	1.0420E+03	5.7460E+02
F29	Avg	3.1292E+03	**3.1000E+03**	**3.1000E+03**	5.2124E+06	3.9229E+03	1.6816E+04	1.1877E+07	3.2854E+07
	Std	2.5153E+01	**0.0000E+00**	**0.0000E+00**	4.4648E+06	9.8477E+01	5.0694E+03	9.2945E+06	3.3032E+07
F30	Avg	3.6979E+03	**3.2000E+03**	7.0859E+03	1.6570E+04	5.3510E+03	3.1312E+04	3.9380E+05	1.4993E+04
	Std	1.5326E+02	**0.0000E+00**	1.0098E+04	4.5285E+03	4.1068E+02	8.8926E+03	1.1559E+05	6.9942E+03

**Table 7:** The p value of ABHGS with involved improved algorithms

Function	ABHGS	WLSSA	CLSGMFO	OBLGWO	WDE	IGWO	SCADE	CBA
F1	∼	8.4570E-01	**1.9531E-03**	**1.9531E-03**	**1.9531E-03**	**1.9531E-03**	**1.9531E-03**	1.0000E+00
F2	∼	**1.9531E-03**	**1.9531E-03**	**1.9531E-03**	**1.9531E-03**	**1.9531E-03**	**1.9531E-03**	**1.9531E-03**
F3	∼	**1.9531E-03**	**1.9531E-03**	**1.9531E-03**	**3.9063E-03**	**1.9531E-03**	**1.9531E-03**	**1.9531E-03**
F4	∼	**3.9063E-03**	6.4453E-02	**1.9531E-03**	4.3164E-01	**1.9531E-03**	**1.9531E-03**	**3.9063E-03**
F5	∼	6.9531E-01	1.9336E-01	**1.9531E-03**	**1.9531E-03**	**1.9531E-03**	**1.9531E-03**	**2.**3242E**-01**
F6	∼	1.3086E-01	**2.7344E-02**	**2.7344E-02**	1.3086E-01	**4.8828E-02**	**1.9531E-03**	**1.9531E-03**
F7	∼	1.6016E-01	3.2227E-01	**1.9531E-03**	**5.8594E-03**	**1.9531E-03**	**1.9531E-03**	8.4570E-01
F8	∼	**1.9531E-03**	**1.9531E-03**	**1.9531E-03**	**1.9531E-03**	**1.9531E-03**	**1.9531E-03**	**1.9531E-03**
F9	∼	**1.9531E-03**	**1.9531E-03**	**1.9531E-03**	3.2227E-01	**9.7656E-03**	**1.9531E-03**	**1.9531E-03**
F10	∼	**1.9531E-03**	**1.9531E-03**	**1.9531E-03**	**1.9531E-03**	**1.9531E-03**	**1.9531E-03**	**1.9531E-03**
F11	∼	**1.9531E-03**	**1.9531E-03**	**1.9531E-03**	1.9336E-01	**1.9531E-03**	**1.9531E-03**	**1.9531E-03**
F12	∼	1.3086E-01	**1.9531E-03**	**1.9531E-03**	**3.9063E-03**	**1.9531E-03**	**1.9531E-03**	**1.9531E-03**
F13	∼	**1.9531E-03**	**1.9531E-03**	**1.9531E-03**	**1.3672E-02**	**1.9531E-03**	**1.9531E-03**	**1.9531E-03**
F14	∼	**4.8828E-02**	**1.9531E-03**	**1.9531E-03**	**4.8828E-02**	**9.7656E-03**	**1.9531E-03**	**1.9531E-03**
F15	∼	**4.8828E-02**	6.4453E-02	**3.9063E-03**	**1.9531E-03**	**1.9531E-03**	**1.9531E-03**	**1.9531E-03**
F16	∼	**1.9531E-03**	**1.9531E-03**	**1.9531E-03**	**3.9063E-03**	**3.9063E-03**	**1.9531E-03**	**1.9531E-03**
F17	∼	**5.8594E-03**	8.3984E-02	6.4453E-02	**1.9531E-03**	5.5664E-01	**1.9531E-03**	**5.8594E-03**
F18	∼	1.3086E-01	**1.9531E-03**	**1.9531E-03**	**1.9531E-03**	**1.9531E-03**	**1.9531E-03**	**9.7656E-03**
F19	∼	**1.9531E-03**	**1.9531E-03**	**1.9531E-03**	5.5664E-01	**1.9531E-03**	**1.9531E-03**	**1.9531E-03**
F20	∼	3.7500E-01	**9.7656E-03**	**1.9531E-02**	**1.9531E-03**	2.3242E-01	**1.9531E-03**	1.0000E+00
F21	∼	3.7500E-01	6.9531E-01	**1.3672E-02**	**1.9531E-03**	2.3242E-01	**1.9531E-03**	1.3086E-01
F22	∼	2.3242E-01	**4.8828E-02**	6.4453E-02	**3.7109E-02**	7.6953E-01	**1.9531E-03**	**1.9531E-03**
F23	∼	1.0000E+00	1.0000E+00	**1.9531E-03**	**1.9531E-03**	**1.9531E-03**	1.0000E+00	**1.9531E-03**
F24	∼	**1.5625E-02**	**1.5625E-02**	**1.5625E-02**	**1.9531E-03**	**1.9531E-03**	**1.5625E-02**	**1.9531E-03**
F25	∼	1.0000E+00	1.0000E+00	1.0000E+00	**1.9531E-03**	**1.9531E-03**	1.0000E+00	**1.9531E-03**
F26	∼	2.7539E-01	1.9336E-01	**3.7109E-02**	**5.8594E-03**	**3.9063E-03**	**1.9531E-03**	6.4453E-02
F27	∼	1.0000E+00	1.0000E+00	5.0000E-01	**1.9531E-03**	**1.9531E-03**	**1.5625E-02**	**1.9531E-03**
F28	∼	1.0000E+00	1.0000E+00	6.2500E-02	**1.9531E-03**	**1.9531E-03**	**7.8125E-03**	**1.9531E-03**
F29	∼	**1.9531E-03**	**1.9531E-03**	**1.9531E-03**	**1.9531E-03**	**1.9531E-03**	**3.9063E-03**	**1.9531E-03**
F30	∼	**1.9531E-03**	4.3164E-01	**1.9531E-03**	**1.9531E-03**	**1.9531E-03**	**1.9531E-03**	**1.9531E-03**
+/−/ =	∼	11/5/14	15/3/12	24/1/5	16/9/5	26/0/4	27/1/2	23/1/6
ARV	**1.9**	3.2	3.5	5.6	2.7	5.466667	7.166667	5.6
Rank	**1**	3	4	6	2	5	8	6

**Figure 9:**
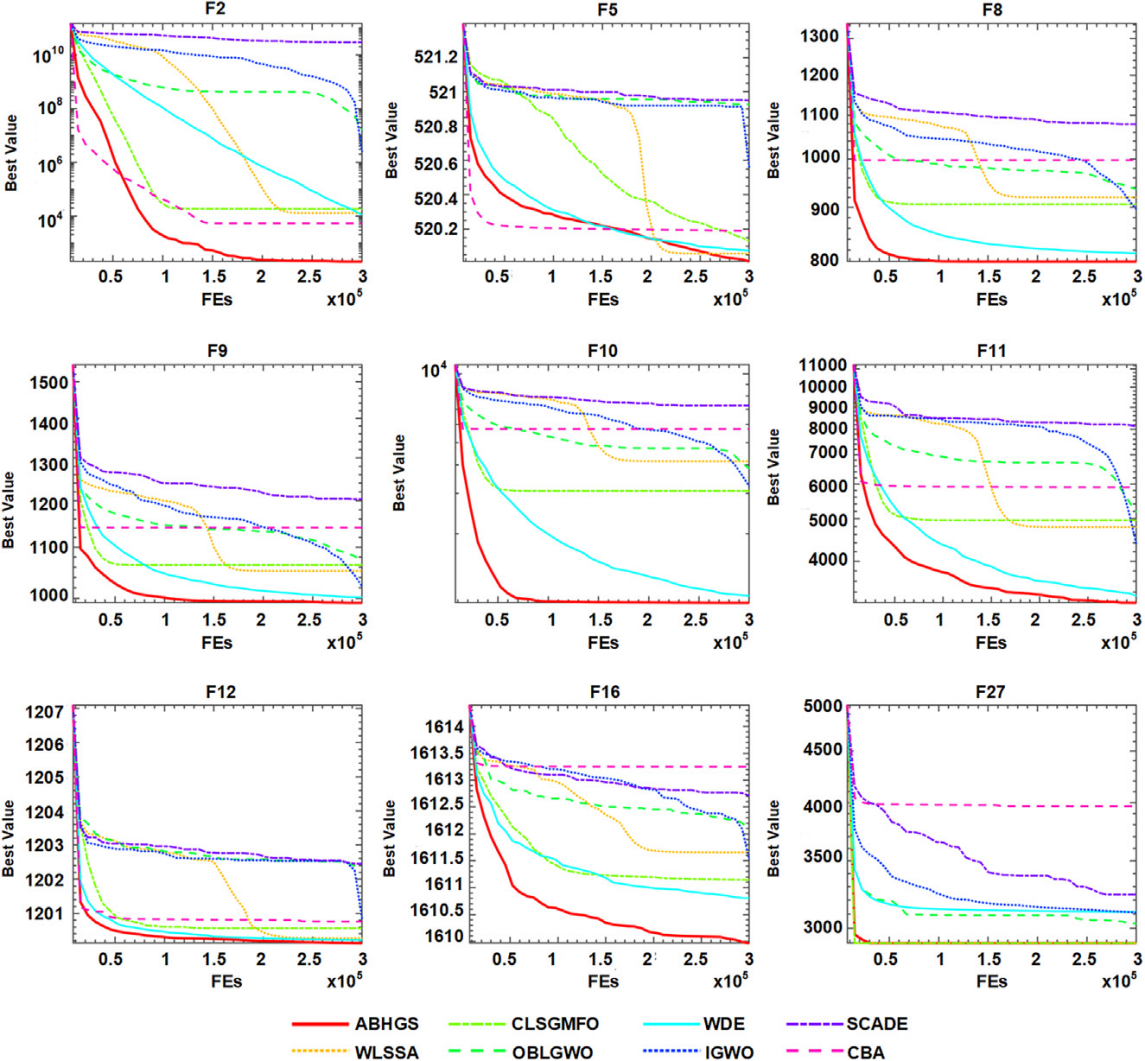
Convergence curves of the modified methods on CEC 2017 functions

The convergence curves of advanced methods on 30 CEC 2017 functions are presented in Figure 9. The curves of F2, F5, F8, F9, F10, F11, F12, F16, and F27 of ABHGS converge faster than other advanced algorithms except for CBA. However, CBA converges prematurely on these test functions and falls into local optimal solutions. Meanwhile, the proposed method has the best fitness, proving that ABHGS has a strong global search ability and gets rid of local optima. Therefore, ABHGS can produce optimal solutions accurately.

In a word, ABHGS optimizes different types of functions on the CEC 2017 functions, outperforming some newly reported advanced techniques. The combination of artificial bee colony strategy and Gaussian bare-bone structure to the HGS algorithm enables the method to achieve a higher quality solution in the optimization process, making the local exploitation and global exploration in a better equilibrium state.

### Feature selection

This section transfers the above continuous ABHGS to a discrete binary version for feature selection. We found the most suitable transfer function for ABHGS and compared it with other optimizers. Fourteen high-dimensional gene datasets are used in this study. The detailed characteristics of these high-dimensional gene datasets are displayed in Table 8. Besides, leave-one-out cross-validation is an effective method for feature selection in the datasets; that is, one sample in the dataset is taken as the test set to prove the classification accuracy of the classifier, and the others are used as the training set. Each dataset’s validation number equals the number of test datasets. This feature selection task is conducted on the KNN classifier, whose field size k is equal to 1.

**Table 8:** The characteristics of 14 high-dimensional gene datasets

Datasets	Samples	features	Categories
Brain_Tumor1	90	5920	5
Brain_Tumor2	50	10,367	4
CNS	60	7130	2
Colon	62	2000	2
DLBCL	77	5470	4
Leukemia	72	7131	2
Leukemia1	72	5328	5
Leukemia2	72	11,225	3
Lung_Cancer	203	12,601	3
Prostate_Tumor	102	10,509	2
SRBCT	83	2309	4
Tumors_9	60	5726	9
Tumors_11	174	12,533	11
Tumors_14	308	15,009	26

Ensuring that the same dataset, settings, and metrics are used when comparing AI techniques is critical to ensure an accurate comparison.^101^^,^^102^ Different datasets can have varying levels of complexity, which could lead to inaccurate results.^103^^,^^104^ Additionally, different metrics can measure performance in different ways, making it difficult to compare results between AI techniques.^105^^,^^106^ Using the same dataset and metrics for comparison ensures that the results are reliable and valid.^107^ For fairness, all the algorithms are run on the same primary function with the same random initialization. The parameters of the main function and involved algorithms are set in Table 9. The number of search agents is set to 20. The fold is set to 10, and the maximum number of evaluations is set to 50. The entire algorithms are implemented in the same software and hardware environment.

**Table 9:** Parameter settings for the six comparison algorithms

Method	Reference	Common parameters	Parameters
bHHO	Heidari et al.^43^	Dimension = Number of attributes; population size = 20;	∼
bSMA	Kumar et al.^130^	Dimension = Number of attributes; population size = 20;	z=0.03
bAOA	Hashim et al.^35^	Dimension = Number of attributes; population size = 20;	a=5
bHGS	Yang et al.^69^	Dimension = Number of attributes; population size = 20;	l=0.08; LH=100
bINFO	Ahmadianfar et al.^41^	Dimension = Number of attributes; population size = 20;	c=2;d=4
bRUN	Ahmadianfar et al.^39^	Dimension = Number of attributes; population size = 20;	ɡ=[02]
bABC	Zorarpacı et al.^131^	Dimension = Number of attributes; population size = 20;	Limit=300;Foodnumber=15
bWOA	Mirjalili and Lewis^66^	Dimension = Number of attributes; population size = 20;	$a_{1}=\left[0\;2\right];a_{2}=\left[-2-1\right];b=1$

To prove the effectiveness of the bABHGS_KNN model for feature selection, it is compared with the discrete versions of WOA, SMA, HGS, HHO, RUN, AOA, INFO, and ABC. The parameter settings for the eight comparison algorithms are displayed in Table 9. Compared with the binary MAs, the value of the nearest neighbor in KNN is set to 1 in this study. The 14 dataset’s primary data is normalized to −1 and 1 at the beginning. The entire experiment is conducted in the same environmental conditions. Based on the machine learning literature,^108^^,^^109^^,^^110^^,^^111^ 10-fold cross-validation (CV) analysis is adopted to classify fair and objective results.

Tables 10, 11, 12, and 13 describe the statistical outcomes of the 14 high-dimensional gene datasets simulated by intelligent swarm algorithms. The minimum value for each dataset is bolded. Table 10 shows the MAs' average error rate, and ABHGS wins the smallest error rate value in each dataset. Therefore, the bABHGS method ranks number one and shows superior performance in terms of error rate, followed by bHHO, bHGS, bWOA, bABC, bSMA, bINFO, bAOA, and bRUN. The number of selected features is displayed in Table 11, and bABHGS has the smallest number of selected features on 14 high-dimensional gene datasets. In a word, bABHGS is far more competitive than the other optimizers in reducing the features. The best fitness results from the significant measurements are presented in Table 12. The fitness combines classification accuracy and the number of features the objective function accumulates to assess the selected features. Most of the data in bold are from the bABHGS method, which shows its excellent performance on the high-dimensional gene dataset.

**Table 10:** The comparison average error rate results of involved swarm intelligent optimizers

Datasets	Metrics	bABHGS	bWOA	bSMA	bHGS	bHHO	bRUN	bABC	bAOA	bINFO
Brain_Tumor1	std	**0**	**0**	0.0632	0.0316	**0**	0.0877	0.0316	0.0717	0.0446
	avg	**0**	**0**	0.0200	0.0100	**0**	0.0881	0.0100	0.0511	0.0211
Brain_Tumor2	std	**0**	**0**	**0**	**0**	**0**	0.2048	**0**	0.1315	0.0527
	avg	**0**	**0**	**0**	**0**	**0**	0.21	**0**	0.0567	0.0167
CNS	std	**0**	0.0452	0.0655	**0**	**0**	0.2164	**0**	0.0833	0.0452
	avg	**0**	0.0143	0.0310	**0**	**0**	0.2629	**0**	0.0643	0.0143
Colon	std	**0**	**0**	**0**	**0**	**0**	0.1246	0.0527	0.0843	0.0655
	avg	**0**	**0**	**0**	**0**	**0**	0.2714	0.0167	0.0976	0.0310
DLBCL	std	**0**	**0**	**0**	**0**	**0**	0.1249	**0**	**0**	**0**
	avg	**0**	**0**	**0**	**0**	**0**	0.1161	**0**	**0**	**0**
Leukemia	std	**0**	**0**	**0**	**0**	**0**	0.0778	**0**	**0**	**0**
	avg	**0**	**0**	**0**	**0**	**0**	0.0726	**0**	**0**	**0**
Leukemia1	std	**0**	**0**	**0**	**0**	**0**	0.0983	**0**	**0**	**0**
	avg	**0**	**0**	**0**	**0**	**0**	0.0554	**0**	**0**	**0**
Leukemia2	std	**0**	**0**	**0**	**0**	**0**	0.0602	**0**	**0**	**0**
	avg	**0**	**0**	**0**	**0**	**0**	0.0286	**0**	**0**	**0**
Lung_Cancer	std	**0**	**0**	0.0235	0.0158	0.0158	0.0457	0.0151	0.0235	0.0201
	avg	**0**	**0**	0.0145	0.0050	0.0050	0.0591	0.0048	0.0145	0.0095
Prostate_Tumor	std	**0**	**0**	0.0316	0.0287	**0**	0.1580	0.0316	0.0527	0.0422
	avg	**0**	**0**	0.0100	0.0091	**0**	0.1391	0.0100	0.0500	0.0200
SRBCT	std	**0**	**0**	**0**	**0**	**0**	0.0809	**0**	**0**	**0**
	avg	**0**	**0**	**0**	**0**	**0**	0.0583	**0**	**0**	**0**
Tumors_9	std	**0**	0.0703	0.0527	0.0351	**0**	0.3094	0.0395	**0**	0.0351
	avg	**0**	0.0222	0.0167	0.0111	**0**	0.3067	0.0125	**0**	0.0111
Tumors_11	std	0.0186	0.0425	0.01756	0.0416	0.0333	0.0822	0.0378	0.0436	**0.0176**
	avg	0.0059	0.0289	**0.0056**	0.0369	0.0105	0.0835	0.0395	0.0512	0.0056
Tumors_14	std	**0.0328**	0.0667	0.0527	0.0576	0.0730	0.0817	0.0611	0.0950	0.0426
	avg	**0.2122**	0.2500	0.2924	0.2449	0.2359	0.3292	0.2924	0.2656	0.2457
Rank-ARV		**1.1429**	2.3571	3.3571	2.3571	1.5000	9.0000	3.2143	4.7857	3.7857
Rank		**1**	3	6	3	2	9	5	8	7

**Table 11:** The number of selected feature result of involved swarm intelligent optimizers

Datasets	Metrics	bABHGS	bWOA	bSMA	bHGS	bHHO	bRUN	bABC	bAOA	bINFO
Brain_Tumor1	std	**0.6325**	1.7764	50.7131	0.6750	41.0941	721.5933	5.0651	757.9124	214.3947
	avg	**1.8**	3.6	61.6	3.3	36.5	2217	4.1	678.6	313.8
Brain_Tumor2	std	**0.5164**	1.1005	305.9392	0.6325	49.2275	999.6689	0.7071	505.6728	286.9948
	avg	**1.4**	2.1	235.1	2.8	28.3	4105.8	1.5	637.4	442.3
CNS	std	**0.4216**	0.6667	129.5102	3.0930	18.1184	618.6430	0.8233	1193.7719	158.6401
	avg	**1.2**	2	267	3.3	30.5	2699.4	1.7	1656.3	716
Colon	std	0.5271	**0.4216**	31.8659	1.3540	4.2740	192.6032	1.1005	297.0963	53.3568
	avg	**1.5**	2.2	34.9	2.5	4.4	661.3	1.9	186	74.5
DLBCL	std	0.4216	2.3664	29.4937	0.8756	4.3063	490.7015	**0.3162**	653.5056	103.2862
	avg	1.2	2.6	34.1	1.9	5.9	2113.8	**1.1**	495.4	189.6
Leukemia	std	**0.4831**	1.0541	39.4716	2.4060	3.7431	827.3877	**0.4831**	273.0738	118.7455
	avg	**1.3**	2	53.7	2.3	7.3	2814	**1.3**	514.2	170.6
Leukemia1	std	**0.4216**	1.4142	12.7980	0.9718	19.6424	752.4359	0.8756	182.2081	164.8369
	avg	**1.8**	3	48.3	2.5	20.4	1630.3	1.9	343	242.9
Leukemia2	std	**0.4831**	1.1738	150.2308	0.8433	15.5453	1317.2936	1.1005	485.9552	118.5181
	avg	**1.7**	2.6	93.2	2.6	23.1	4217.3	2.1	551	254.1
Lung_Cancer	std	**10.3864**	28.1111	70.9288	24.7227	119.0920	1391.7347	36.6195	682.4995	444.7305
	avg	**11.9**	19.7	146.3	26.1	114.7	4046.2	35.9	1400.6	744.1
Prostate_Tumor	std	**0.6667**	4.0838	248.7296	125.2140	72.1862	1276.9845	10.0995	1386.1660	464.4055
	avg	**2**	3.7	251.8	45.9	64.2	4243.3	6	1907.2	903.4
SRBCT	std	**0.7379**	2.2136	14.3434	1.3166	4.7714	191.0733	1.1972	130.7578	20.2748
	avg	**2.9**	4.3	33.2	3.8	11.9	858.1	**2.9**	186.4	81.8
Tumors_9	std	31.5998	9.7439	261.3690	333.7594	84.6155	509.7897	**3.8239**	1018.3894	712.0521
	avg	15.9	5.5	413	153.3	99.7	2159.8	**4.2**	1514.8	904.3
Tumors_11	std	128.2950	150.3757	377.1790	175.6904	343.1570	1218.8802	**90.9017**	1842.3418	879.9994
	avg	**164.6**	227.2	602	244.7	537.6	4779.9	249	2927	1860.3
Tumors_14	std	**332.9462**	783.9875	917.9929	435.2029	947.1118	857.3160	787.7948	1989.4100	1384.8884
	avg	**511.6**	738	1235	681.3	2279.7	6272.7	690.4	6258.4	3674.5
Rank-ARV		**1.2143**	3.0000	5.9286	3.3571	5.0000	9.0000	2.2857	8.0000	7.0000
Rank		**1**	3	6	4	5	9	2	8	7

**Table 12:** The comparison of best fitness values of involved optimizers

Datasets	Metrics	bABHGS	bWOA	bSMA	bHGS	bHHO	bRUN	bABC	bAOA	bINFO
Brain_Tumor1	std	**5.34E-06**	1.50E-05	6.03E-02	3.00E-02	3.47E-04	5.15E-02	3.00E-02	6.61E-02	4.27E-02
	avg	**1.52E-05**	3.04E-05	1.95E-02	9.53E-03	3.08E-04	4.89E-02	9.53E-03	5.43E-02	2.27E-02
Brain_Tumor2	std	**2.49E-06**	5.31E-06	1.48E-03	3.05E-06	2.37E-04	9.84E-02	3.41E-06	1.24E-01	5.04E-02
	avg	**6.75E-06**	1.01E-05	1.13E-03	1.35E-05	1.36E-04	5.75E-02	7.23E-06	5.69E-02	1.80E-02
CNS	std	**2.96E-06**	4.29E-02	6.21E-02	2.17E-05	1.27E-04	7.79E-02	5.77E-06	7.80E-02	4.34E-02
	avg	**8.42E-06**	1.36E-02	3.13E-02	2.31E-05	2.14E-04	7.05E-02	1.19E-05	7.27E-02	1.86E-02
Colon	std	1.32E-05	**1.05E-05**	7.97E-04	3.39E-05	1.07E-04	1.08E-01	5.01E-02	8.22E-02	6.22E-02
	avg	**3.75E-05**	5.50E-05	8.73E-04	6.25E-05	1.10E-04	1.01E-01	1.59E-02	9.74E-02	3.13E-02
DLBCL	std	3.85E-06	2.16E-05	2.70E-04	8.01E-06	3.94E-05	2.96E-03	**2.89E-06**	5.97E-03	9.44E-04
	avg	1.10E-05	2.38E-05	3.12E-04	1.74E-05	5.39E-05	8.40E-03	**1.01E-05**	4.53E-03	1.73E-03
Leukemia	std	**3.39E-06**	7.39E-06	2.77E-04	1.69E-05	2.62E-05	1.71E-03	**3.39E-06**	1.92E-03	8.33E-04
	avg	**9.12E-06**	1.40E-05	3.77E-04	1.61E-05	5.12E-05	6.87E-03	**9.12E-06**	3.61E-03	1.20E-03
Leukemia1	std	**3.96E-06**	1.33E-05	1.20E-04	9.12E-06	1.84E-04	2.43E-03	8.22E-06	1.71E-03	1.55E-03
	avg	**1.69E-05**	2.82E-05	4.53E-04	2.35E-05	1.91E-04	6.78E-03	1.78E-05	3.22E-03	2.28E-03
Leukemia2	std	**2.15E-06**	5.23E-06	6.69E-04	3.76E-06	6.92E-05	2.74E-03	4.90E-06	2.16E-03	5.28E-04
	avg	**7.57E-06**	1.16E-05	4.15E-04	1.16E-05	1.03E-04	7.75E-03	9.35E-06	2.45E-03	1.13E-03
Lung_Cancer	std	**4.12E-05**	1.12E-04	2.23E-02	1.51E-02	1.49E-02	2.00E-02	1.43E-02	2.44E-02	1.89E-02
	avg	**4.72E-05**	7.82E-05	1.44E-02	4.85E-03	5.21E-03	1.73E-02	4.67E-03	1.94E-02	1.20E-02
Prostate_Tumor	std	**3.17E-06**	1.94E-05	3.04E-02	2.73E-02	3.43E-04	4.54E-02	3.00E-02	4.90E-02	4.00E-02
	avg	**9.52E-06**	1.76E-05	1.07E-02	8.85E-03	3.05E-04	4.01E-02	9.53E-03	5.66E-02	2.33E-02
SRBCT	std	**1.60E-05**	4.80E-05	3.11E-04	2.85E-05	1.03E-04	2.55E-03	2.59E-05	2.83E-03	4.39E-04
	avg	**6.28E-05**	9.32E-05	7.19E-04	8.23E-05	2.58E-04	6.91E-03	**6.28E-05**	4.04E-03	1.77E-03
Tumors_9	std	**2.76E-04**	6.68E-02	5.04E-02	3.33E-02	7.39E-04	1.08E-01	3.76E-02	8.89E-03	3.57E-02
	avg	**1.39E-04**	2.12E-02	1.94E-02	1.19E-02	8.71E-04	5.93E-02	1.19E-02	1.32E-02	1.85E-02
Tumors_11	std	1.75E-02	4.04E-02	**1.67E-02**	3.98E-02	3.14E-02	4.51E-02	3.60E-02	4.29E-02	1.94E-02
	avg	**6.24E-03**	2.83E-02	7.68E-03	3.60E-02	1.21E-02	5.10E-02	3.86E-02	6.04E-02	1.27E-02
Tumors_14	std	**3.18E-02**	6.12E-02	4.95E-02	5.47E-02	6.89E-02	7.27E-02	5.68E-02	8.99E-02	3.87E-02
	avg	**2.03E-01**	2.40E-01	2.82E-01	2.35E-01	2.32E-01	2.75E-01	2.80E-01	2.73E-01	2.46E-01
Rank-ARV		**1.0714**	3.6429	6.0714	3.5714	4.0000	8.5000	3.3571	8.0000	6.4286
Rank		**1**	4	6	3	5	9	2	8	7

**Table 13:** The comparison time cost of involved optimizers

Datasets	Metrics	bABHGS	bWOA	bSMA	bHGS	bHHO	bRUN	bABC	bAOA	bINFO
Brain_Tumor1	std	2.5252	0.2973	0.7852	**0.2194**	0.5155	0.6119	0.6045	0.3511	0.6536
	avg	98.5572	12.129	19.9034	**10.4247**	18.5947	24.6148	13.4641	22.0087	16.2854
Brain_Tumor2	std	1.8348	0.4336	0.4864	**0.1649**	0.6308	0.6271	0.7832	0.4371	0.5704
	avg	157.0758	13.2214	23.8736	**11.6293**	19.3058	28.3172	13.3329	21.5483	17.9986
CNS	std	2.1229	0.3084	0.4434	**0.1921**	0.2957	0.3860	0.4036	0.5892	0.3860
	avg	114.7253	9.2040	18.0425	**8.8127**	14.7480	20.8028	12.3730	17.4893	15.4106
Colon	std	0.6908	**0.0727**	0.2635	0.1054	0.1792	0.1101	0.1318	0.0839	0.0977
	avg	23.8107	**2.7985**	5.6089	2.9552	5.8581	6.5536	2.9477	3.4795	3.1960
DLBCL	std	0.7943	0.3086	0.3540	0.3527	**0.2566**	0.4094	0.5975	0.4729	0.4142
	avg	109.1352	**8.3326**	14.9278	8.6467	14.4141	21.0766	12.2371	17.1504	13.4058
Leukemia	std	1.5137	**0.2404**	0.3348	0.3062	0.4172	0.4489	0.5314	0.3679	0.5580
	avg	79.6747	10.8370	18.1801	**10.5893**	18.3265	23.7089	11.3907	18.4572	15.7804
Leukemia1	std	1.6070	0.2740	0.3662	**0.2300**	0.3154	0.4428	0.4841	0.4402	0.3296
	avg	92.3648	**7.9214**	15.4914	7.9647	13.8235	18.2597	9.5742	15.9516	12.1072
Leukemia2	std	2.5865	0.5991	**0.2744**	0.4047	0.4879	0.5995	0.8183	0.5613	1.0232
	avg	181.6542	17.4971	28.5760	**16.4472**	28.2665	39.5341	17.1282	30.6693	19.7165
Lung_Cancer	std	2.7267	1.4924	1.2712	1.5133	2.3843	3.9889	**0.7967**	2.3543	3.2919
	avg	265.2328	42.9970	58.3429	49.6144	113.0123	181.9989	**39.4170**	122.6448	70.3598
Prostate_Tumor	std	3.0749	0.6269	0.7078	**0.3855**	0.8532	1.8712	1.4075	0.6790	0.8021
	avg	181.3939	20.6688	31.9412	**19.8780**	35.8911	52.6291	22.9948	40.2175	25.7981
SRBCT	std	0.7306	**0.1024**	0.2267	0.1667	0.1922	0.4555	0.2904	0.1790	0.1132
	avg	55.4933	**4.0875**	8.0258	4.7642	8.5251	12.8495	4.5333	5.8635	5.0219
Tumors_9	std	1.2445	0.2163	**0.1911**	0.3130	0.3019	0.8290	0.4772	0.3920	0.4053
	avg	108.4235	**7.2428**	15.2858	7.3478	12.7310	17.4270	8.8244	12.7556	8.9935
Tumors_11	std	2.8575	1.6779	1.2035	1.7806	2.3108	6.2901	**0.6951**	1.9645	1.3460
	avg	264.9391	35.9078	52.9967	42.4763	94.5031	142.9292	**30.7897**	95.1537	68.5403
Tumors_14	std	8.0631	3.2855	4.1388	3.7524	11.3731	12.6566	**1.8083**	7.9564	9.0488
	avg	418.1131	106.0704	150.2631	108.0340	265.8921	433.5448	**70.0437**	282.5614	204.2613
Rank-ARV		8.9286	**1.7143**	5.6429	1.9286	5.5714	8.0714	2.3571	6.5000	4.2857
Rank		9	**1**	6	2	5	8	3	7	4

**Figure 10:**
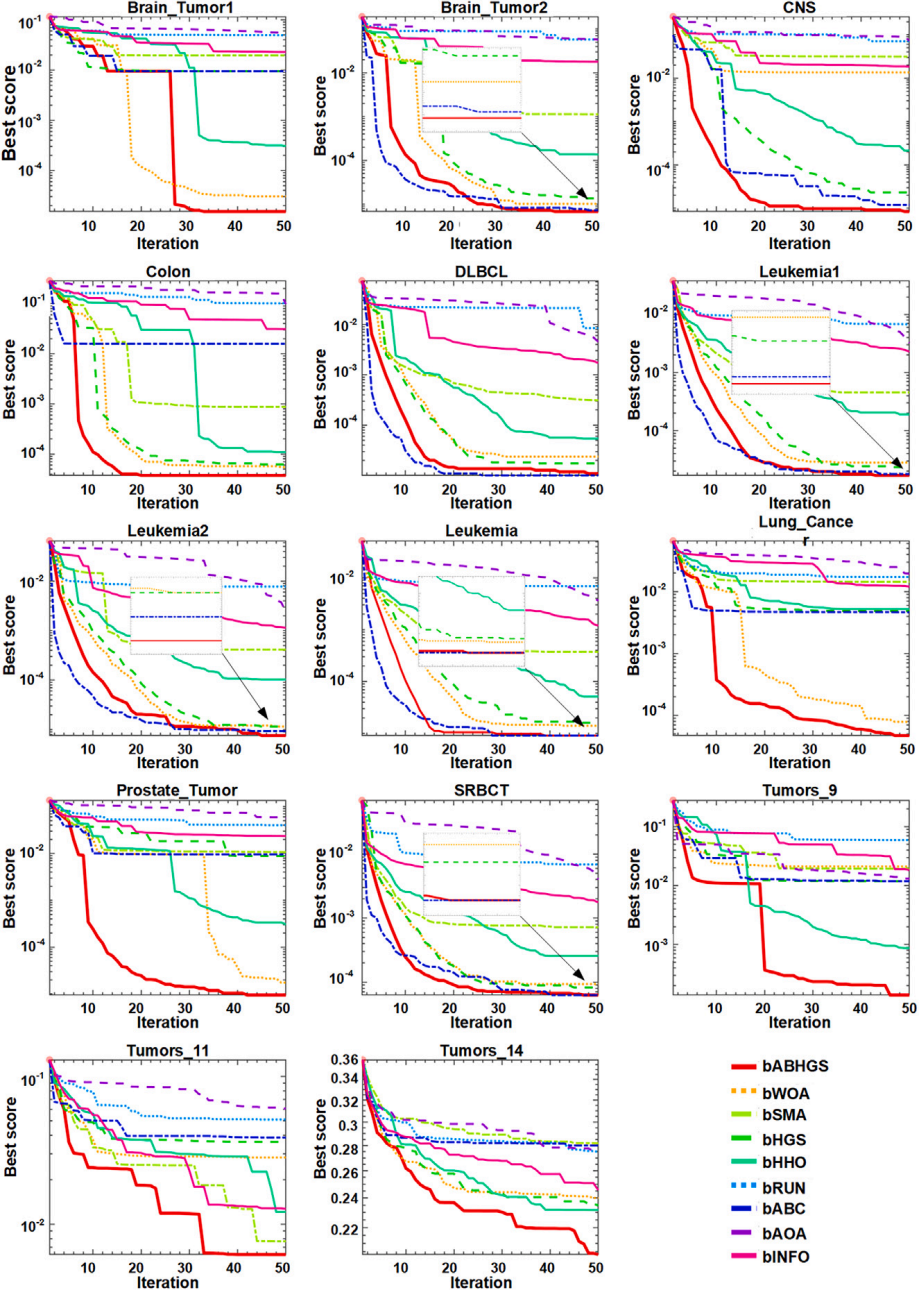
The comparison of best fitness values of 8 optimizers for 14 high-dimensional gene datasets

As the average values and std values shown in Tables 10, 11, and 12, the bABHGS method has excellent performance, satisfactory average fitness, and minimal SD in all 14 high-dimensional gene datasets, which performs more stable than other involved optimizers. It can be seen from Table 13 that the average calculation time results of bABHGS are low-ranking, showing the high complexity of the method. It takes more time cost due to the enhancement of the performance. The artificial bee colony strategy and Gaussian bare-bone structure impact the increased time cost. Figure 10 presents the fitness convergence curve of 9 algorithms for 14 high-dimensional gene datasets. The bABHGS-KNN model gains the best fitness value on 14 high-dimensional gene datasets, ensuring its diversity through strong search ability.

Compared with other optimizers, bABHGS is the best regarding error rate, number of features, and fitness on 14 high-dimensional gene datasets. Through the calculation time, the cost of the proposed method is not ideal; bABHGS can choose the optimal subset on most microarray datasets in terms of the optimal fitness and the minimal classification error rate without losing the meaningful features. Simulation results prove that the combination of the artificial bee colony strategy and Gaussian bare-bone structure to HGS guarantees its global exploration. Therefore, the proposed method achieves a more effective equilibrium between local exploitation search and global exploration search. In future work, the proposed method can also be applied to more cases, such as the optimization of machine learning models,^112^ MRI reconstruction,^113^ fine-grained alignment,^114^ location-based services,^115^^,^^116^ Alzheimer’s disease identification,^117^ renewable energy generation,^118^ information retrieval services,^119^^,^^120^^,^^121^^,^^122^ power distribution network,^123^ medical signals,^124^^,^^125^^,^^126^ and iris or retinal vessel segmentation.^127^^,^^128^

### Limitations of the study

The present study has several limitations. First, the impact of each method on HGS is not evaluated in feature selection task trials. A preliminary test compares ABHGS, AHGS, BHGS, and HGS on CEC 2017 benchmark functions. Additionally, in-depth assessments of each strategy’s influence on ABHGS may be examined. Second, the experimental simulation and validation portion is insufficient owing to the absence of a comparison with more enhanced algorithms. Our suggested bABHGS is only compared to basic types, with no hybrid method. Third, It is evident from the experiments that the algorithm is hampered by a long execution time. To address this issue, incorporating parallel computing into the algorithm could be an option.

### Conclusions and future works

This article introduces the artificial bee colony strategy and Gaussian bare-bone structure to HGS, namely ABHGS. To validate the global search ability of ABHGS, the simulation is conducted on CEC 2017 functions. Besides, we design experiments to show the excellent performance of ABHGS, such as history trajectory analysis, balance analysis, function test, and feature selection. ABHGS compares against the original algorithm, other conventional algorithms, and advanced methods. The experimental results demonstrate that ABHGS performs better than competing metaheuristic algorithms due to the enhanced equilibrium between exploration and exploitation propensities. Then we implement a discrete binary ABHGS method to select a vital subset and combine it with KNN classifier for 14 publicly high-dimensional gene datasets in this article. The results demonstrate the superiority of the proposed method in terms of average error rate, average fitness value, and the number of feature subsets. Although the time cost of bABHGS is higher than other methods because of the performance improvement. In conclusion, the bABHGS-KNN model can achieve higher classification accuracy and select fewer features, showing excellent performance for high-dimensional gene data feature selection problems.

This model will be further developed for accuracy and stability in future work. We intend to apply the bABHGS method to other practical high-dimensional datasets. Meanwhile, the proposed method can be used in more fields, such as the parameter identification of photovoltaics, engineering optimization, financial prediction, and disease diagnosis. Finally, we can develop an Artificial Intelligence framework for most feature selection problems.

## STAR★Methods

### Key resources table

**Table:** 

REAGENT or RESOURCE	SOURCE	IDENTIFIER
**Software and algorithms**		

HGS algorithm	Ali Asghar Heidari	https://aliasgharheidari.com/HGS.html
ABHGS algorithm	This paper	https://doi.org/10.5281/zenodo.7775014

### Resource availability

#### Lead contact

Further requests for information should be directed and will be handled by the lead contact, Huiling Chen, email: chenhuiling.jlu@gmail.com.

#### Materials availability

This study did not generate new materials.

### Method details

The proposed approach consists of hunger game search, gaussian bare-bone structure, artificial bee colony strategy, K-nearest neighbor classifier, explained in detail in this section.

#### Hunger game search

The HGS method is a population-based metaheuristic algorithm introduced in this section. It imitates the foraging behaviors of social animals driven by hunger and always solves both constrained and unconstrained optimization issues. The continuous HGS with mathematical expressions is introduced in history trajectory section, which is easier for new users to understand.

#### Approaching food

The approaching food behaviors of social animals are elaborated on in this part, updating their positions with each iteration. Social animals cooperate in hunting prey in groups, but the possibility exists that some individuals do not take part in teamwork. Thus hunger-driven behaviors of HGS approaching food are expressed as Equation (6).

(Equation 6) $$\vec{X^{t+1}}=\left\{ \begin{array}{cc} \vec{X^{t}}\cdot \left(1+randn\left(1\right)\right)\text{,} & r_{1}<l\\ \vec{W_{1}}\cdot \vec{X_{b}}+\vec{R}\cdot \vec{W_{2}}\cdot \left|\vec{X_{b}}-\vec{X^{t}}\right|\text{,} & r_{1}>l,r_{2}>E\\ \vec{W_{1}}\cdot \vec{X_{b}}-\vec{R}\cdot \vec{W_{2}}\cdot \left|\vec{X_{b}}-\vec{X^{t}}\right|\text{,} & {r_{1}>l,r}_{2}<E \end{array}\right.$$

where $\vec{X^{t+1}}$ does the current position update a new vector; $\vec{X^{t}}$ denotes the position vector of individuals in current iteration; randn(1) indicates a random number which satisfies normal distribution; both $\vec{W_{1}}$ and $\vec{W_{2}}$ represent independent weights of hunger; $\vec{R}$ means the vector in the range of [−a,a] ,which is calculated as Equation (7); $\vec{X_{b}}$ is one best position in the individual; $r_{1}$ and $r_{2}$ are two random numbers between 0 and 1, respectively, and l represents a parameter.

(Equation 7) $$\vec{R}=2\times a\times rand-a$$

(Equation 8) $$a=2\times \left(1-\frac{t}{Maxiter}\right)$$

where t represents the current iteration, and Maxiter means the maximum number of iterations; rand stands for a random vector that number is in the range of [0,1]. A variation control E is defined as follows Equation (9).

(Equation 9) E=sech(|F(i)−BF|)

where F(i) denotes the fitness value of the *i-*th agent and BF stands for the best fitness value of agents in the process. The hyperbolic function Sech is expressed in Equation (10).

(Equation 10) $$\mathrm{sech}\left(x\right)=\frac{2}{e^{x}+e^{-x}}$$

As Equation (6) shows, three instructions are divided into two aspects for the overall situation, namely $\vec{X}$-based and $\vec{X_{b}}$-based. The early game focuses on independent hunting behavior, with few agents working together as a team. The late two games imitate cooperative foraging behavior with $\vec{W_{1}}$, $\vec{R}$, and $\vec{W_{2}}$. These different locations explore the optimal solution in the search space.

#### Hunger role

The hunger-driven behaviors of agents are expressed in mathematical formulas in this part.

The expression for $\vec{W_{1}}\left(i\right)$ is shown as Equation (11) and $\vec{W_{2}}\left(i\right)$ can be computed as Equation (12).

(Equation 11) $$\vec{W_{1}}\left(i\right)=\left\{ \begin{array}{cc} hungry\left(i\right)\cdot \frac{N}{SHungry}\times r_{4}\text{,} & r_{3}<l\\ 1 & r_{3}>l \end{array}\right.$$

(Equation 12) $$\vec{W_{2}}\left(i\right)=\left(1-\exp\left(-\left|hungry\left(i\right)-SHungry\right|\right)\right)\times r_{5}\times 2$$

where hungry(i) denotes the hunger level of each individual; N represents the number of agents; SHungry is sum(hungry), which calculated by the hungry amount of the whole individuals; $r_{3}$ indicates a random value in the range of [0,1]; both $r_{4}$ and $r_{5}$ are random numbers between 0 and 1. The hungry(i) is shown as Equation (13).

(Equation 13) $$hungry\left(i\right)=\left\{ \begin{array}{cc} 0\text{,} & F\left(i\right)==BF\\ hungry\left(i\right)+H\text{,} & F\left(i\right)!=BF \end{array}\right.$$

where the conventional hunger level adds a new hunger sensation (H) to create population’s diversity; BF is the fitness value of the best agent; F(i) is the fitness of *i-*th agent. The expression of H is shown as follows:

(Equation 14) $$TH=\frac{F\left(i\right)-BF}{WF-BF}\times r_{6}\times 2\times \left(UB-LB\right)$$

(Equation 15) $$H=\left\{ \begin{array}{cc} LH\times \left(1+r\right)\text{,} & TH<LH\\ TH\text{,} & TH\geq LH \end{array}\right.$$

where $r_{6}$ is a random number ranging in [0,1]; WF is the fitness of the worst individual; UB indicates the upper bound in the search space, LB denotes the lower boundary, and hunger H has a lower bound LH.

A brief description of the HGS optimizer is given above, which provides a simple and efficient mathematical model that can be applied to continuous optimization problems. The pseudo-code of the continuous HGS is shown as Algorithm 2.

Algorithm 2 **Pseudo-code of hunger games search (HGS)**

 Initialize the parameters N,Maxiter,l,t,D(dimension),SHungry;

 Initialize the positions of Individuals $X_{i}$(i=1,2,⋯,N);

 
**While** (t≤Maxiter)

 Calculate the fitness of all Individuals;

 Sort the fitness of all Individuals;

 Update $BF,WF,X_{b}$;

 Set Hungry by Equation (13)

 Set the $W_{1}$ by Equation (11);

 Set the $W_{2}$ by Equation (12)

 
**For** eachindividual

 Compute E by Equation (9);

 Calculate R by Equation (7)

 Update the position by Equation (6);

 
**End** For

 
t=t+1;

 
**End While**

 
**Return** $BF,X_{b}$

#### Gaussian bare-bone structure

In the HGS algorithm, when food shortage events occur, it forces some agents to a new region to forage. A Gaussian bare-bone mechanism was first proposed by Kennedy, inspired by the distribution histogram of PSO after 1,000,000 iterations.^81^ The particle’s velocity was removed through Gaussian distribution, and its position in the next iteration was updated. In the barebones PSO algorithm (BBPSO), the following formula is applied to replace the location of the *i*-th individual.

(Equation 16) $$X_{i,j}^{t+1}=N\left(\begin{array}{cc} \frac{{pbest}_{i,j}^{t}+X_{b,j}}{2}\text{,} & \left|{pbest}_{i,j}^{t}-X_{b,j}\right| \end{array}\right)$$

where $X_{i,j}^{t+1}$ stands for the position of the *i-*th individual in the *j*-th dimension in the (t+1) iteration; N(·) is a Gaussian distribution function; ${pbest}_{i,j}^{t}$ is the optimum location of the *i*-th individual in the *j*-th dimension currently; $X_{b,j}$ denotes the *j*-th dimension of the global optimum location in the population; $\frac{{pbest}_{i,j}^{t}+X_{b,j}}{2}$ is the arithmetic mean value; $\left|{pbest}_{i,j}^{t}-X_{b,j}\right|$ is the absolute value function of variance. Gaussian barebone is shown as Equation (17).

Equation (17) $$X_{i,j}^{t+1}=\left\{ \begin{array}{ccc} N\left(\frac{{pbest}_{i,j}^{t}+X_{b,j}}{2},\left|{pbest}_{i,j}^{t}-X_{b,j}\right|\right)\text{,} & rand<CR & \left(1\right)\\ {pbest}_{i,j}^{t}+k\times \left(X_{k_{1},j}^{t}-X_{k_{2},j}^{t}\right)\text{,} & rand\geq CR & \left(2\right) \end{array}\right.$$

where k is a number randomly selected between 0 and 1, the indices $k_{1}$ and $k_{2}$ represent two different indices derived from the population set 1,2,…,N*,* which differ from i. rand is a number ranging in [0,1], and the scale factor CR is to control the difference vector. The pseudo-code of the Gaussian bare-bone structure is presented as Algorithm 3.

Algorithm 3 **Pseudo-code of Gaussian bare-bone structure**

 **INPUT:** The search agent population $X^{t}$, the optimum location of the *i*-th individual ${pbest}_{i}^{t}$

 
**For** i=1toN

 
**For** i=jtodim

 
**if** rand<CR

 Update the position $X_{i,j}^{t+1}$ by Equation 17 (1)

 
**else**

 Update the position $X_{i,j}^{t+1}$ by Equation 17 (1);

 
**End if**

 
**End For**

 Bring back search agent population $X_{i}^{t+1}$ going outside;

 Calculate the fitness of all Individuals;

 Update search agent population $X_{i}^{t+1}$ by greedy selection;

 Update the optimum location of the *i*-th individual ${pbest}_{i}^{t+1}$ by greedy selection;

 
**End For**

 **Return t**he updated $X^{t+1}\;and\;{pbest}_{i}^{t+1}$

#### Artificial bee colony strategy

An artificial bee colony (ABC) was proposed by Karaboga in 2005^82^ based on the foraging behavior of bee colonies. The employed bees comprise the group’s first half, while the onlookers' bees comprise the second. The number of hired bees is equal to the number of optimal solutions. This paper has four steps in the search process of the ABC strategy.

(1) A hired bee modifies the position of food sources (solutions) in memory based on local information (visual information) and finds nearby food sources, then assesses the quality of the food. In ABC, the search for adjacent food sources is expressed by Equation (18).

(Equation 18) $${food}_{i,j}^{t+1}={food}_{i,j}^{t}+{\vec{r}}_{i}^{j}\left({food}_{i,j}^{t}-{food}_{k,j}^{t}\right)$$

where ${food}_{i,j}^{t+1}$ is the candidate food source stands for the position of the *i*th individual in the *j*th dimension in the (t+1) iteration and ${food}_{i,j}^{t}$ stands for the position in the t iteration; ${\vec{r}}_{i}^{j}$ represents a random vector which value ranging from [−1,1], and ${food}_{k,j}^{t}$ denotes a selected solution k that is different from i.

(2) Once the foraging bees have completed their search, they communicate to the onlooker bees in the dance area the quantity of nectar and its source. This is a trait of ABC artificial bee colonies. The onlookers assess the nectar data from all foragers and select a food source based on probability, which is determined by Equation 19 according to the fitness values of each solution.

(Equation 19) $$prob\left(i\right)=0.9\times \frac{\min\left\{F\left(1\right),F\left(2\right),\ldots ,F\left(i\right),\ldots ,F\left(N\right)\right\}}{F\left(i\right)}+0.1$$

(3) A random number between 0 and 1 is generated for each source in the ABC. Suppose the probability value prob(i) associated with that source, as stated in Equation (14), is higher than the random number. In that case, the onlooker bee modifies the position of this food source site, similar to what happens with hired bees. After evaluating the source, a greedy selection mechanism is used; the onlooker bee either remembers the new position or retains the old one.
(4) After the entire hired bees and onlooker bees finish their searches in a cycle, a new food source position is created. For every location of a new food source, a random population $X_{c}$ is selected from the population gained by HGS. After being evaluated, the random population selected either memorizes the food source’s new position or keeps the old one by greedy selection. Randomly selected population $X_{c}$ are generated by the following formula.

(Equation 20) C=randi([1,N])

where $X_{c}$ stands for a randomly chosen solution C.

The pseudo-code of the artificial bee colony strategy is shown in Algorithm 4.

Algorithm 4 **Pseudo-code of artificial bee colony strategy**

 **INPUT：** The search agent population $X^{t}$, the food source position ${food}^{t}$

 
**For** i=1toN/2

 Update the position of the food source ${food}_{i}^{t+1}$ by Equation (18);

 Bring back the position of food source ${food}_{i}^{t+1}$ going outside;

 Calculate the fitness of all food source ${food}_{i}^{t+1}$;

 Select the better solution between ${food}_{i}^{t+1}$ and ${food}_{i}^{t}$ as a new food source position;

 
**End For**

 Calculate *prob* by Equation (19);

 Initialize the parameters i,it;

 
**While** (it<N/2)

 
**if** (rand<prob(i))

 
it=it+1;

 Update the position of the food source ${food}_{i}^{t+1}$ by Equation (18);

 Bring back the position of food source ${food}_{i}^{t+1}$ going outside;

 Calculate the fitness of all food source ${food}_{i}^{t+1}$;

 Select the better solution between ${food}_{i}^{t+1}$ and ${food}_{i}^{t}$ as new ${food}_{i}^{t+1}$

 
**end if**

 Update i;

 
**End While**

 
**For** i=1toN/2

 Update C by Eq. (20);

 Update position $X_{c}^{t+1}$ by greedy selection in food source ${food}_{i}^{t+1}$ and position $X_{c}^{t}$;

 Select the better solution between ${food}_{i}^{t+1}$ and $X_{c}^{t}$ as new $X_{c}^{t+1}$;

 
**End For**

 **Return** The updated $X^{t+1}$ and ${food}^{t+1}$

#### K-Nearest Neighbor Classifier

When working with huge datasets, various classifiers like SVM and ANN have reported delayed convergence and being time-consuming.^83^ In contrast to these conventional training methods, neural networks with random weights, such as the bidirectional stochastic configuration, demonstrate low training complexity, good performance, and quick speed. However, applying it directly to complicated problems is challenging due to its low complexity. Additionally, the thin network architecture causes numeric problems when working with massive datasets.^84^

On the other hand, The K-Nearest Neighbor (KNN)^85^ was a simple, non-parametric, and effective learning technique that achieved excellent performance in function classification and approximation problems with a completion rate and high classification accuracy.^86^^,^^87^^,^^88^ In recent research, KNN also shows excellent performance in training speed and classification accuracy,^89^^,^^90^ so this paper adopts K nearest neighbor (KNN) as a classifier for experimental evaluation. It is an instance-based learning model which predicts the class of a new instance according to the majority vote of the k-nearest neighbor class. The minimal distance between the new instance and the training points is used to decide the new instance’s class based on the similarity measurement. The similarity is used in the literature most and is measured by the Euclidean distance. The Euclidean distance calculation procedure for two D-dimensional points $Z_{1}$ and $Z_{2}$ is shown as follows:

(Equation 21) $$Distance\;\left(Z_{1},Z_{2}\right)={{\left(\sum_{i=1}^{D}(z_{1}^{i}-z_{2}^{i}\right)}^{2})}^{\frac{1}{2}}$$

Due to fast training speed, easy implementation, and excellent efficiency, this paper uses a KNN classifier to evaluate the classification accuracy.

#### Proposed methodology

##### ABHGS algorithm for global optimization

There are various variants of HGS because the original HGS has some drawbacks that may miss some promising regions and cause population stagnation. To overcome this problem, a new HGS with an artificial bee strategy and Gaussian bare-bone structure is proposed, namely ABHGS. It introduces the artificial bee colony strategy and Gaussian bare-bone structure to the original HGS. In the ABHGS, two cooperative mechanisms provide diversity to the population and improve the objective function’s feasibility and convergence capacity. The artificial bee colony strategy contributes to global exploration, and the Gaussian bare-bone structure enhances local exploitation. Therefore, ABHGS can maintain diverse search abilities and meet the population’s needs in a certain evolutionary stage under limited computing resources. The detailed pseudo-code of ABHGS is presented in Algorithm 5. In a word, it is easier to understand the dynamic, fitness-wise optimizer. The flowchart of ABHGS is shown in Figure S1.

Algorithm 5 **The proposed ABHGS**

 Initialize the parameters N,Maxiter,l,D,SHungry,t;

 Initialize the positions of Individuals $X_{i}$(i=1,2,⋯,N);

 Initialize the food source position ${food}_{i}$;

 **While** (t ≤Maxiter)

 Update the positions of Individuals $X_{i}$ by Algorithm 2;

 Sort the fitness of all Individuals;

 Update $BF,WF,X_{b},{pbest}_{i}^{t}$;

 Set Hungry by Equation (13);

 Set the $W_{1}$ by Equation (11);

 Set the $W_{2}$ by Equation (12);

 
**For** eachindividual

 Compute E by Equation (9);

 Calculate R by Equation (7);

 Update the position by Equation (6);

 
**End** For

 Update the positions of Individuals $X_{i}$ and food source position ${food}_{i}$ by Algorithm 3;

 
t=t+1;

 
**End While**

 
**Return** $BF,X_{b}$

The computational complexity of ABHGS consists of initialization, an artificial bee colony strategy, and a Gaussian bare-bone structure. N is the number of individuals in the population. T represents the maximal iterations, and D means the dimension. The time complexity of initialization is O(N). The computational sorting complexity is O(NlogN). The complexity of fitness evaluation and hunger update are O(N×D). Meanwhile, the computational complexity of HGS is O(N×(1+T×N×(2+logN+2×D)). The time complexity of the artificial bee colony mechanism is O(T×N) and the computational complexity of the Gaussian bare-bone structure is O(N×D×T). In conclusion, the whole computation complexity of the ABHGS method is O(N×(1+T×N×(3+logN+3×D)). It is not hard to understand that the time cost of ABHGS will be more as the mechanism is added. Therefore, to find the optimal solution, it is a logical side effect that the ABHGS method will obtain a longer to find the optimal solution.

#### Binary ABHGS for feature selection

ABHGS is a modified continuous version of HGS, the discrete binary ABHGS selects crucial features. The artificial bee colony strategy and Gaussian bare-bone structure get useful feature information and generate the most suitable feature selection solution, improving classification accuracy. Since feature selection is a binary problem, its solution is restricted to the binary space {0, 1}. In a word, feature selection based on ABHGS should adopt a binary format. In the optimization process, an n-dimensional vector can stand for a solution whose length is the number of features in the dataset. The solution value can be “0” or “1”, where “0” represents that the feature is not selected and “1” denotes that the feature is chosen. Individuals’ initialization with a binary location vector used a random threshold as Equation (22).

(Equation 22) $$X_{i}^{j}=\left\{ \begin{array}{cc} 0 & rand\leq 0.5\\ 1 & rand>0.5 \end{array}\right.$$

where $X_{i}^{j}$ represents the ith individual in jth dimension of the vector in the population. Then, ABHGS is converted to binary ABHGS (bABHGS), which is implemented by a transfer function (TF). The TF used in this study is presented as Equation (23), and the updating mechanism of positional elements is shown as follows:

(Equation 23) $$T\left(X_{i}^{j}\left(t\right)\right)=\left|\frac{2}{\pi }\arctan\left(\frac{\sqrt{\pi }}{2}X_{i}^{j}\left(t\right)\right)\right|$$

where $X_{i}^{j}\left(t\right)$ represents the ith individual in jth dimension at tth iteration and $X_{i}^{j}\left(t+1\right)$ is the individual of next iteration updated by the conversion formula Equation (24).

(Equation 24) $$X_{i}^{j}\left(t+1\right)=\left\{ \begin{array}{cc} -X_{i}^{j}\left(t+1\right)\text{,} & rand<T\left(x_{i}^{d}\left(t+1\right)\right)\\ X_{i}^{j}\left(t+1\right)\text{,} & rand\geq T\left(x_{i}^{d}\left(t+1\right)\right) \end{array}\right.$$

Feature selection has two contradictory main objectives: the number of selected features and classification accuracy. The higher the classification accuracy, the fewer features are selected, indicating a better classification effect. A fitness function is typically used to assess the quality of each solution throughout the iteration. Finally, bABHGS can balance the classification accuracy and the number of selected features. The resulting adaptive function is as follows:

(Equation 25) $$Fitness=\alpha \cdot \left(1-Acc\right)+\left(1-\alpha \right)\times \left(D_{R}/D\right)$$

where α denotes a weight of classification accuracy ranging in [0,1]; Acc is the classification accuracy; (1−Acc) means the error rate of the classifier; $D_{R}$ is the size of the subset filtered by the optimizer, and D is the total number of features in the dataset. In this study, we set α=0.05 ^91^ from related literature.

Scale the dataset to the range [-1,1] and divide the dataset into training and test sets. This study adopts ten-fold cross-validation. Initializing the population of binary ABHGS, the dimension of the population in bABHGS is the attribute number of the dataset. Then we use the KNN classifier to evaluate the accuracy of selected attributes. It is the bABHGS-KNN model that calculates the fitness of the population using its internal phases. The optimal solution is evaluated and achieved by this model. Finally, an optimal feature subset is an output.

### Quantification and statistical analysis

Detailed description of statistical methods is provided in results and discussion under the following sections: verification of the mechanisms; comparative test with conventional metaheuristic algorithms; comparative test with several modified algorithms; feature selection. The overall experiments are conducted in the same hardware and MATLAB R2018b software environment. In terms of global optimization problem, these methods including ABHGS algorithm evaluated their performance using the statistical average value of the optimal function (Avg) and standard deviation (Std). The smaller the value, the better the performance. If the modification is considered significant statistically, the Wilcoxon signed-rank test is less than 0.05; that is, the p-value is less than 0.05. The Wilcoxon signed-rank test is a non-parametric statistical test at a significance level of 0.05. The Friedman test is a statistical conformance test, too. The symbols “+/=/-” illustrate that the proposed algorithm performs better, equal, or worse than the other comparative method. All statistical details of global optimization are provided in Tables 1, 3, 4, 5, 6, and 7, Figures 4, 6 and 8. In terms of feature selection, datasets size (n) information for all analyses is provided in Table 8. The results are evaluated in terms of classification accuracy, number of selected features, and mean and standard deviation of fitness and run time. Tables 10, 11, 12, and 13 describe the statistical outcomes of the 14 high-dimensional gene datasets simulated by intelligent swarm algorithms. All statistical details are provided and explained in the text.

## Data Availability

• The dataset that informed or guided this study are available online and data reported in this paper will be shared by the lead contact upon request. • All original code generated as part of this study has been deposited at Website: https://aliasgharheidari.com/ or at zenodo, and is publicly available as of the date of publication. A link to code and DOIs are listed in the key resources table. • Any additional information for reanalyzing this work is available from the lead contact upon request.
