# Supplementary material for: An artificial bee bare-bone hunger games search for global optimization and high-dimensional feature selection
Source: iScience. 2023 Apr 21;26(5):106679. doi: 10.1016/j.isci.2023.106679 (PMC10193239; doi:10.1016/j.isci.2023.106679)
Supplement: Document S1. Figure S1 [file mmc1.pdf]

iScience, Volume *26*

Supplemental information

An artificial bee bare-bone hunger games search for global optimization and high-dimensional feature selection

Zhiqing Chen, Ping Xuan, Ali Asghar Heidari, Lei Liu, Chengwen Wu, Huiling Chen, José Escorcia-Gutierrez, and Romany F. Mansour

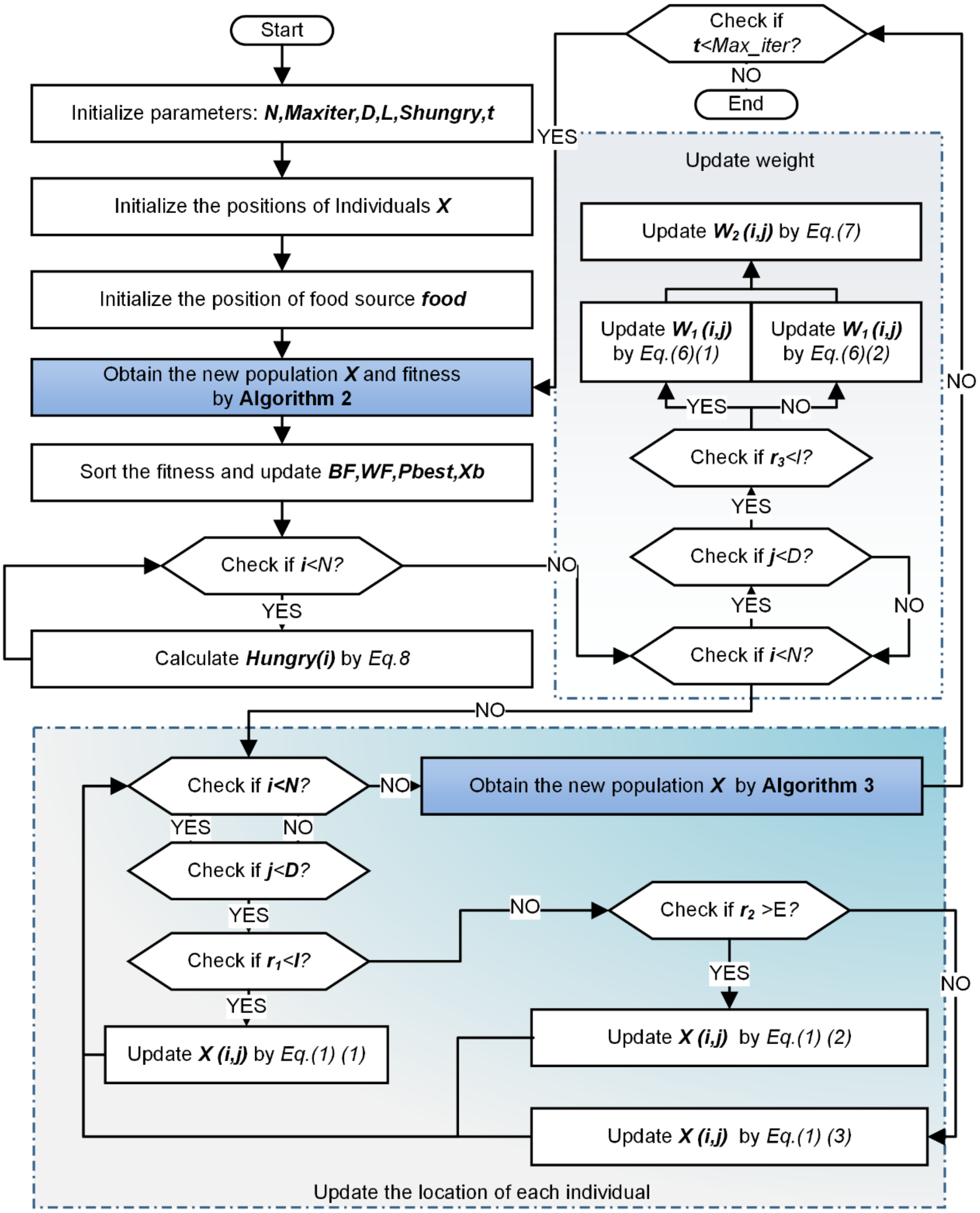

Figure S1. Flowchart of ABHGS,Related to STAR Methods
